# Molecular and Physiological Logics of the Pyruvate-Induced Response of a Novel Transporter in *Bacillus subtilis*

**DOI:** 10.1128/mBio.00976-17

**Published:** 2017-10-03

**Authors:** Teddy Charbonnier, Dominique Le Coq, Stephen McGovern, Magali Calabre, Olivier Delumeau, Stéphane Aymerich, Matthieu Jules

**Affiliations:** a Micalis Institute, INRA, AgroParisTech, Université Paris-Saclay, Jouy-en-Josas, France; b Micalis Institute, INRA, AgroParisTech, CNRS, Université Paris-Saclay, Jouy-en-Josas, France; Tufts University School of Medicine; Harvard University

**Keywords:** *Bacillus subtilis*, LytST, PftA PftB, YsbA YsbB, catabolite repression, malate, pyruvate transport, two-component regulatory systems

## Abstract

At the heart of central carbon metabolism, pyruvate is a pivotal metabolite in all living cells. *Bacillus subtilis* is able to excrete pyruvate as well as to use it as the sole carbon source. We herein reveal that *ysbAB* (renamed *pftAB*), the only operon specifically induced in pyruvate-grown *B. subtilis* cells, encodes a hetero-oligomeric membrane complex which operates as a facilitated transport system specific for pyruvate, thereby defining a novel class of transporter. We demonstrate that the LytST two-component system is responsible for the induction of *pftAB* in the presence of pyruvate by binding of the LytT response regulator to a palindromic region upstream of *pftAB*. We show that both glucose and malate, the preferred carbon sources for *B. subtilis*, trigger the binding of CcpA upstream of *pftAB*, which results in its catabolite repression. However, an additional CcpA-independent mechanism represses *pftAB* in the presence of malate. Screening a genome-wide transposon mutant library, we find that an active malic enzyme replenishing the pyruvate pool is required for this repression. We next reveal that the higher the influx of pyruvate, the stronger the CcpA-independent repression of *pftAB*, which suggests that intracellular pyruvate retroinhibits *pftAB* induction via LytST. Such a retroinhibition challenges the rational design of novel nature-inspired sensors and synthetic switches but undoubtedly offers new possibilities for the development of integrated sensor/controller circuitry. Overall, we provide evidence for a complete system of sensors, feed-forward and feedback controllers that play a major role in environmental growth of *B. subtilis*.

## IMPORTANCE

Pyruvate is a small-molecule metabolite ubiquitous in living cells. Several species also use it as a carbon source as well as excrete it into the environment. The bacterial systems for pyruvate import/export have yet to be discovered. Here, we identified in the model bacterium *Bacillus subtilis* the first import/export system specific for pyruvate, PftAB, which defines a novel class of transporter. In this bacterium, extracellular pyruvate acts as the signal molecule for the LytST two-component system (TCS), which in turn induces expression of PftAB. However, when the pyruvate influx is high, LytST activity is drastically retroinhibited. Such a retroinhibition challenges the rational design of novel nature-inspired sensors and synthetic switches but undoubtedly offers new possibilities for the development of integrated sensor/controller circuitry.

## INTRODUCTION

Several carboxylic acids are substantially secreted by plant roots into the rhizosphere (1). Root exudates are composed of carboxylic acids, such as malate, citrate, and pyruvate, and vary across environments, specifically in response to the presence of phytotoxic compounds (2). Root exudates and released carboxylic acids also enable recruiting beneficial bacteria, such as the Gram-positive model bacterium *Bacillus subtilis*, that help to reduce susceptibility to plant pathogen attack (3–5). The assimilation of carboxylic acids by *B. subtilis* mainly relies on active transport systems, whose expression is induced via two-component systems (e.g., *maeN* by MalK/R [6]) by the transported carbon source (7). Besides carboxylic acids, *B. subtilis* is capable of utilizing a wide variety of carbon sources, including plant materials such as pectin, galactan, polygalacturonan, and rhamnogalacturonan (8–10). Indeed, *B. subtilis* has recently been suggested to be an epiphyte (11). The coassimilation of carbon sources in bacteria is strictly controlled by carbon catabolite repression (CCR). In *B. subtilis*, glucose and malate are the two preferred carbon sources and therefore impose a strict hierarchy for the use of alternative carbon sources (12). At the transcriptional level, the glucose-mediated CCR operates via the master regulator of carbon metabolism CcpA and its cofactors (HPr and Crh) to repress transcription of several targets, among which are the genes encoding the transporters of alternative carbon sources (13). Malate also represses the coutilization of alternative, glycolytic substrates by hijacking the usual glucose-mediated CcpA-dependent catabolite repression (12, 13). Repression occurs upon binding of CcpA in complex with the serine-phosphorylated HPr (P-Ser-HPr) or Crh (P-Ser-Crh) to regions in promoters called catabolite responsive elements (*cre* sites). The carbon-specific gene regulatory networks together with the global mechanism of catabolite repression can be viewed as sensors, feed-forward and feedback controllers that tightly adapt the overall metabolism to changing environments.

Pyruvate is the simplest of the alpha-keto acids and a key metabolite for living cells as the end product of glycolysis, a major substrate for oxidative metabolism, and a branching point for glucose, lactate, acetate, fatty acid, and amino acid syntheses. Because it is at the junction of several essential pathways in both eukaryotic and prokaryotic cells, tight control of its homeostasis and fate is crucial to ensure cell structural stability and robustness to changing environmental growth conditions. In eukaryotes, the mitochondrial enzymes that metabolize pyruvate are physically separated from the cytosolic pyruvate pool and rely on a transport system to shuttle pyruvate across the inner mitochondrial membrane. This transport system consists of a hetero-oligomeric complex composed of carriers MPC1 and MPC2 (14, 15). In prokaryotes, knowledge about pyruvate uptake systems is scarce. To date, two monocarboxylate transport systems with low affinity for pyruvate have been identified and characterized, MctC in *Corynebacterium glutamicum* (16) and MctP in *Rhizobium leguminosarum* (17). For these two systems, the uptake of pyruvate is driven by the electrochemical proton potential, as opposed to a facilitated diffusion, where the energy is provided by the concentration gradient of the substance transported. Although *B. subtilis* is able to grow on pyruvate as the sole carbon source (7), no clear homolog of any pyruvate transporter was found in its genome (18). Recently, the *ysbA* and *lytS* genes were shown to be essential for pyruvate utilization in *B. subtilis* (19). YsbA and LytS present homology to an antiholin-like protein and to a two-component system (TCS) sensor kinase, respectively. The *ysbA* gene is induced in the presence of extracellular pyruvate and transcribed in an operon with the *ysbB* (encoding a putative holin-like protein) gene (20). Immediately upstream from *ysbAB* on the *B. subtilis* chromosome, the *lytS* and *lytT* genes (encoding a putative TCS response regulator) are constitutively transcribed as an operon. van den Esker et al. showed that the deletion of *lytS* abolishes the expression of *ysbA*, which indicates a direct or indirect regulatory role of the putative TCS LytST (19). In addition, the level of induction of *ysbA* in the presence of both pyruvate and glucose is significantly reduced (19).

*B. subtilis* is a long-time model organism (21), and a highly detailed genome sequence, along with transcriptome- and proteome-wide responses to a hundred environmental conditions have been determined (18, 20, 22). Gene essentiality has also been investigated, and libraries of nonessential genes and intervals are freely available (23–25). However, about one-fourth of its genome codes for poorly characterized or completely unknown functions. In the present work, we identified the first bacterial transporter specific for pyruvate, which is a hetero-oligomeric membrane complex composed of YsbA and YsbB and operating as a facilitated transporter. For the sake of clarity, we renamed *ysbAB pftAB* for *p*yruvate-*f*acilitated *t*ransporter. We examined the regulation of *pftAB* by LytST and CcpA in depth and revealed that malate represses *pftAB* by an additional, CcpA-independent mechanism. Although the extracellular pyruvate activates the LytST TCS, we discovered that when the pyruvate influx is high, LytST activity is drastically retroinhibited.

## RESULTS

### Deletion of *pftA* and *pftB* drastically reduces growth on pyruvate.

**FIG 1:**
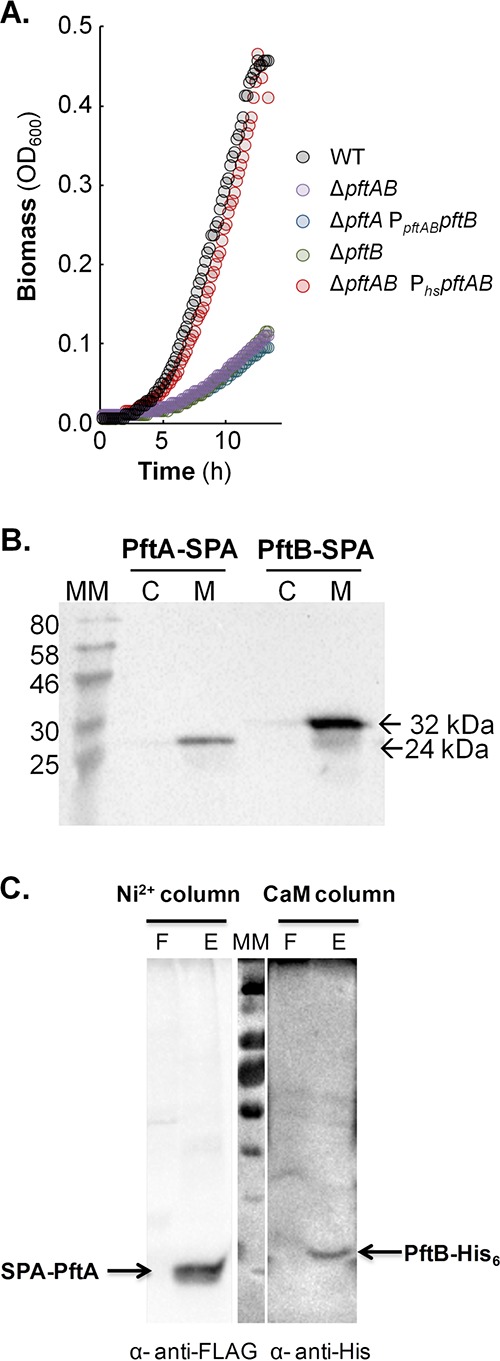
Role and localization of *pftAB*. (A) Growth of the WT, Δ*pftA* P*_pftAB_pftB*, Δ*pftB*, Δ*pftAB*, and Δ*pftAB* P*_hs_pftAB* strains on M9P. (B) Cytoplasmic (C) versus membrane (M) localization of PftA-SPA (24 kDa) and PftB-SPA (32 kDa). Cells were grown in M9SE+P. Western blotting was performed using an anti-FLAG monoclonal antibody as the primary antibody and horseradish peroxidase-conjugated anti-mouse antibody as the secondary antibody. The positions of molecular mass markers (in kilodaltons) are indicated to the left of the gel. (C) Copurification of a N-terminal SPA-tagged PftA and of a C-terminal His-tagged version of PftB. The membrane fraction was first loaded onto a Ni^2+^ column to capture PftB-His_6_, and a Western blot using anti-FLAG antibodies revealed the presence of copurified SPA-PftA (F and E stand for flowthrough and eluate, respectively). The eluate was next loaded onto a CaM column to capture SPA-PftA, and a Western blot using anti-His antibodies revealed the presence of copurified PftB-His. A representative experiment is presented in each panel.

The *pftA* gene (formerly known as *ysbA*) is known to be essential for growth on pyruvate (19). In order to check whether *pftB* (formerly *ysbB*), the gene downstream of *pftA* within the same operon, is essential for growth of *B. subtilis* on pyruvate, we constructed single and double knockout (KO) mutant strains of *pftA* and *pftB* (Table 1). To circumvent a polar effect of the *pftA* KO mutation on the expression of *pftB*, we reinserted upstream of *pftB* the native P_*pftA*_ promoter sequence (Table 1). The deletion of either *pftA*, *pftB*, or *pftAB* resulted in similar, severely impaired growth on pyruvate (M9 medium [12, 13] with pyruvate [M9P]) (Fig. 1A). These strains did not show any phenotype different from that of the wild-type (WT) strain when grown on other substrates (e.g., glucose [M9 medium with glucose {M9G}], malate [M9 medium with malate {M9M}], and glutamate and succinate [M9 medium with glutamate and succinate {M9SE}] [see Table S1 in the supplemental material]). Complementation by an isopropyl-β-d-thiogalactopyranoside (IPTG)-inducible *pftAB* cassette (P*_hs_pftAB*) inserted at the *amyE* locus restored growth on M9P (Fig. 1A; Table S1) to the WT level (0.25 h^−1^). Although we did not observe a strict essentiality of *pftA* for cell growth on pyruvate, our findings are consistent with that of van den Esker et al. (19) and additionally demonstrated that *pftB* is necessary for growth on pyruvate. This suggested that *pftA* and *pftB* gene products may operate in concert.

**TABLE 1:** *B. subtilis* and *C. glutamicum* strains used in this study

Strain	Relevant genotype	Reference(s), source, or construction^a^
*B. subtilis* strains		
BSB168	Wild type (prototroph)	20, 22
GM2924	Δ*crh*::*aphA3*	13
GM2933	*ptsH1~cat*	13
GM1619	Δ*malS*::*phleo ytsJ*::pMUTIN2 (*erm*) *maeA*::*aphA3 trpC2*	Laboratory collection
GM2907	*ccpA*::Tn*917*Δ(*term lacZ*)::*phleo*	Laboratory collection
GM1626	*ywkB*::pMUTIN (*erm*) *trpC2*	Laboratory collection
TC01	*maeA′*::[pMUTIN2 Δ(*lacZ*-*ery*)::*kan*]	GM1619 → BSB168
TC03	*ywkB*::pMUTIN2 (*erm*)	GM1626 → BSB168
TC28	*maeA′*::[pMUTIN2 Δ(*lacZ*-*ery*)::*kan*] P*_hs_malS/cm*	pDR111_*malS* → TC01
TC29	P*_hs_malS/cm*	pDR111_*malS* → BSB168
TC35	P*_pftAB_-gfpmut3/spec*	pBSB_P_pftAB_ → BSB168
TC36	*ccpA*::Tn*617*Δ(*term lacZ*)::*phleo* P*_pftAB_-gfpmut3/spec*	pBSB_P_pftAB_ → GM2907
TC58	Δ*lytST*::*cm*	pGEMT_*lytST*::*cm* → BSB168
TC59	Δ*lytST*::*cm* P*_pftAB_-gfpmut3/spec*	pBSB_P_pftAB_ → TC58
TC60	Δ*pftAB*::*cm*	pGEMT_*pftAB*::*cm* → BSB168
TC61	Δ*pftB*::*cm*	pGEMT_*pftB*::*cm* → BSB168
TC62	Δ*pftA*::*cm* P*_pftAB_pftB*	pGEMT *___pftA*::*cm*_P_*pftAB*_ → BSB168
TC63	P*_pftAB_-lacZ/cm*	pDG1661_P_*pftAB*_ → BSB168
TC64	*ccpA*::Tn*917*Δ(*erm lacZ*)::*phleo* P*_pftAB_-lacZ/cm*	TC63 → GM2907
TC73	*lytST*::*cm* P*_hs_lytST/erm*	pMUTIN4_*lytST* → TC58
TC74	Δ*pftAB*::*cm amyE*::P*_hs_pftAB/spec*	pDR111_*pftAB* → TC60
TC75	Δ*pftB*::*cm amyE*::P*_hs_pftAB/spec*	pDR111_*pftAB* → TC61
TC76	Δ*pftA*::*cm* P*_pftAB_pftB amyE*::P*_hs_pftAB*	pDR111_*pftAB* → TC62
TC86	*ccpA*::Tn9*17*Δ(*erm lacZ*)::*phleo maeA′*::[pMUTIN2 Δ(*lacZ*-*ery*)::*kan*]	TC01 → GM2907
TC87	*ccpA*::Tn9*17*Δ(*erm lacZ*)::*phleo ywkB*::pMUTIN2 (*erm*)	TC03 → GM2907
TC88	*maeA′*::[pMUTIN2 Δ(*lacZ*-*ery*)::*kan*] P*_pftAB_-gfpmut3/spec*	TC01 → TC35
TC89	*ywkB*::pMUTIN2 (*erm*) P*_pftAB_-gfpmut3/spec*	TC03 → TC35
TC90	*ccpA*::Tn*917*Δ(*erm lacZ*)::*phleo maeA′*::[pMUTIN2 Δ(*lacZ*-*ery*)::*kan*] P*_pftAB_-gfpmut3/spec*	TC01 → TC36
TC91	*ccpA*::Tn*917*Δ(*erm lacZ*)::*phleo ywkB*::pMUTIN2 (*erm*) P*_pftAB_-gfpmut3/spec*	TC03 → TC36
TC99	Δ*crh*::*aphA3 ptsH1~cat*	GM2925 → GM2933
TC100	Δ*crh*::*aphA3* P*_pftAB_-gfpmut3/spec*	GM2925 → TC35
TC101	*ptsH1~cat* P*_pftAB_-gfpmut3/spec*	TC35 → GM2933
TC102	Δ*crh*::*aphA3 ptsH1~cat* P*_pftAB_-gfpmut3/spec*	GM2924 → TC101
TC103	*ccpA*::Tn*917*Δ(*erm lacZ*)::*phleo maeA′*::[pMUTIN2 Δ(*lacZ*-*ery*)::*kan*] *P_hs_malS/cm*	TC29 → TC86
TC104	*maeA′*::[pMUTIN2 Δ(*lacZ*-*ery*)::*kan*] P*_hs_malS/cm* P*_pftAB_-gfpmut3/spec*	TC29 → TC88
TC105	*ccpA*::Tn*617Δ*(*term lacZ*)::*phleo maeA′*::[pMUTIN2 Δ(*lacZ*-*ery*)::*kan*] P*_hs_malS/cm* P*_pftAB_-gfpmut3/spec*	TC29 → TC90
TC112	Δ*pftAB*::*cm* P*_pftAB_-gfpmut3/spec*	TC35 → TC60
TC113	Δ*pftB*::*cm* P*_pftAB_-gfpmut3/spec*	TC35 → TC61
TC114	Δ*pftA*::P*_pftAB_-pftB/cm* P*_pftAB_-gfpmut3/spec*	TC35 → TC62
TC122	P_*pftAB*_* pftA*-*SPA*/*erm*	pPftA_SPA → BSB168
TC124	P_*pftAB*_* pftB*-*SPA*/*erm*	pPftB_SPA → BSB168
TC125	P_*pftAB-lytT2*_-*gfpmut3*/*spec*	pBSB_P_*pftAB-lytT2*_ → BSB168
TC126	P_*pftAB-lytT1*_._*2*_*-gfpmut3*/*spec*	pBSB_P_*pftAB-lytT1*_._*2*_ → BSB168
TC132	*amyE*::P*_hs_pftAB/spec*	pDR111_*pftAB* → BSB168
TC136	*amyE*::P*_hs_pftAB/spec* P*_pftAB_-gfpmut3/cm*	pBSBIX_P_*pftAB*_ → TC132
TC149	P_*pftAB-lytT1*_-*gfpmut3*/*spec*	pBSB_P_*pftAB-lytT1*_ → BSB168
TC150	Δ*pftAB*::*cm* P_*pftAB-lytT1*_/*spec*	TC60 → TC149
TC151	Δ*pftAB*::*cm* P_*pftAB-lytT1*_,_*2*_/*spec*	TC60 → TC126
TC152	Δ*pftAB*::*cm* P_*pftAB-lytT2*_/*spec*	TC60 → TC125
TC153	Δ*pftAB*::*cm* P_*hs*_*pftAB*/*erm* P*_pftAB_-gfpmut3/spec*	pDG1664_P*_hs_pftAB* → TC112
TC154	Δ*pftAB*::*cm* P_*hs*_*pftAB*/*erm*	pDG1664_P*_hs_pftAB* → TC60
TC163	*ccpA*::Tn9*17*Δ(*term lacZ*)::*phleo pftAB*::*cm*	TC60 → GM2907
TC164	*ccpA*::Tn9*17*Δ(*term lacZ*)::*phleo pftAB*::*cm* P*_pftAB_-gfpmut3/spec*	TC60 → TC133
TC166	*ccpA*::Tn917*Δ*(*term lacZ*)::*phleo pftAB*::*cm* P_*pftAB-lytT1*_,*_2_/spec*	TC60 → TC147
TC175	P_*hs*_P_*pftAB*_*-gfpmut3/spec*	pDR111_P_hs_P_pftAB_ → BSB168
TC178	*ccpA*::Tn917*Δ*(*term lacZ*)::*phleo* P_*hs*_P_*pftAB*_*-gfpmut3/spec*	GM2907 → TC175
TC199	*amyE*::P_*xyl*_ *SPA*-*pftA pftB*-*His/spec*	pSG-SPA-NTER → BSB168

*C. glutamicum* strains		
ATCC 130TC	Wild type	16
Cg0953	Δ*mctC*	16
TC200	Wild type	pXmj19 → ATCC 13032
TC201	Δ*mctC*	pXmj19 → Cg0953
TC202	P_*tac*_-*pftAB/cm*	pXmj_pftAB → ATCC 13032
TC203	Δ*mctC* P_*tac*_-*pftAB/cm*	pXmj_pftAB → Cg0953

a Arrows indicate construction by transformation.

10.1128/mBio.00976-17.8 TABLE S1  Growth rates of wild-type, mutant, and complemented strains in M9 plus various substrates. Download TABLE S1, PDF file, 0.01 MB. Copyright © 2017 Charbonnier et al. 2017 Charbonnier et al. This content is distributed under the terms of the Creative Commons Attribution 4.0 International license.

### PftA and PftB form a membrane protein complex.

Both PftA and PftB were predicted to be membrane proteins (Fig. S1A) (19). To validate their localization, we constructed *B. subtilis* cells expressing C-terminal sequential peptide affinity (SPA)-tagged PftA (PftA-SPA) and SPA-tagged PftB (PftB-SPA) and analyzed samples corresponding to the cytosolic and membrane fractions by Western blotting using anti-FLAG antibodies. The results showed that both PftA-SPA and PftB-SPA were mainly, if not exclusively, present in the membrane fraction (Fig. 1B). We next asked whether PftA and PftB form a protein complex in the membrane. We performed tandem affinity purification (TAP) using strains expressing the SPA-tagged PftA and PftB proteins and appropriate controls. Strains were grown in M9P, and the purified (PftA-SPA and PftB-SPA) and copurified proteins were identified using mass spectrometry after tryptic or tryptic/chymotryptic digestions. PftB was specifically detected in the sample containing SPA-tagged PftA (Fig. S1B), which suggests that PftA and PftB form a complex in the membrane. However, mass spectrometry failed to detect PftA in the tandem affinity-purified sample containing SPA-tagged PftB due to the fact that the trypsin and trypsin/chymotrypsin digestions of PftA generated only poorly detected peptides.

10.1128/mBio.00976-17.2 FIG S1  Predicted structure and verified interaction of PftA and PftB at the membrane. (A) PftA and PftB were predicted to be membrane proteins by using the TMHMM program (49), and the predicted structures were visualized with the Protter software (http://wlab.ethz.ch/protter/start/) (50). The predictions are in good agreement, although Protter and TMHMM differ in the number of predicted transmembrane domains for PftB, i.e., with 7 and 5 predicted, respectively. Each blue number represents a putative transmembrane domain. (B) Tandem affinity purification (TAP) of PftA-SPA. The quantity of PftB that is copurified with PftA-SPA (strain TC122 [Table 1]) is given based on MS spectral counting (Text S1). For a control, we applied a mock tandem affinity purification with a membrane preparation from *B*. *subtilis* TC74, a strain overexpressing the *pftA pftB* operon (Text S1). The results of a representative experiment are presented. PftB was specifically detected in the sample containing PftA-SPA, which suggests that PftA and PftB form a complex in the membrane. In contrast, in the mock purification realized with the strain expressing *pftA* and *pftB* with 50 µM IPTG, traces of PftB could be detected but no PftA. Assuming that the number of MS spectra reflects crudely the quantity of protein present in a sample (47), the quantity of PftB was eightfold higher in the PftA-SPA purification than in the mock purification. Download FIG S1, TIF file, 0.1 MB. Copyright © 2017 Charbonnier et al. 2017 Charbonnier et al. This content is distributed under the terms of the Creative Commons Attribution 4.0 International license.

To fully demonstrate that PftA and PftB form a membrane complex, a new construct was made which allowed easy detection of PftA. A N-terminal SPA-tagged PftA along with a C-terminal His-tagged version of PftB were assembled. The SPA-*pftA* and *pftB*-His_6_ synthetic genes were inserted at the *amyE* locus under the control of the xylose-inducible promoter, P_*xyl*_. The expression of SPA-PftA and PftB-His_6_ was induced for 3 h during exponential growth. The membrane fraction was first loaded onto a Ni^2+^ column to capture PftB-His_6_. Western blotting using anti-FLAG antibodies revealed the presence of copurified SPA-PftA (Fig. 1C, Ni^2+^ column, lane E). The eluate was consecutively loaded onto a CaM column to capture SPA-PftA. A Western blot using anti-His antibodies was performed, and copurified PftB-His_6_ was detected (Fig. 1C, CaM column, lane E). In addition, the flowthrough fractions of the first and second columns did not show the presence of any of the two partner proteins, indicating that the complex is stable. The two-step purification experiment was also conducted swapping the order of the columns, and identical results were obtained (not shown). These results indicated that PftA and PftB form a hetero-oligomeric membrane protein complex.

### The PftAB complex operates as a pyruvate transporter.

**FIG 2:**
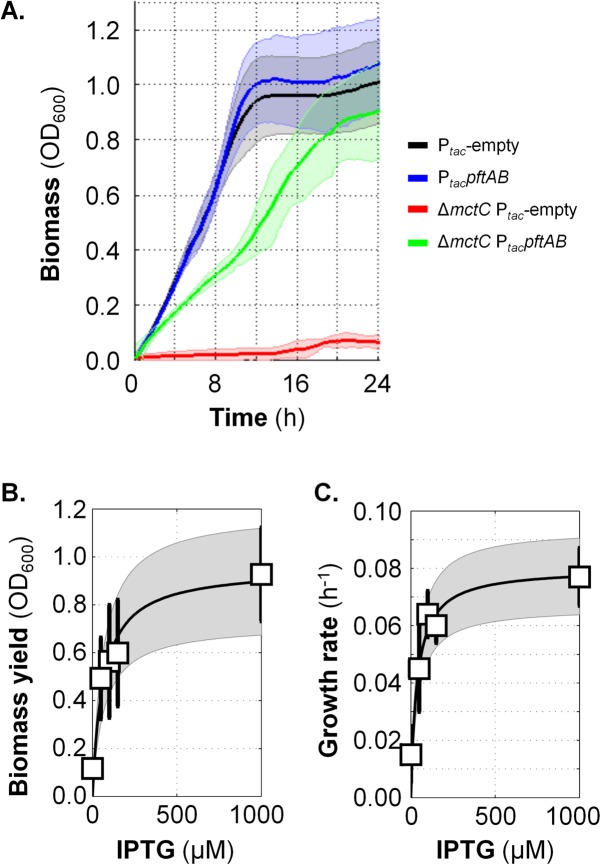
Functional complementation of the pyruvate uptake-deficient Δ*mctC* mutant of *C. glutamicum* by PftAB. (A) Growth in MM1 medium plus pyruvate (MM1+P) of the WT and Δ*mctC C. glutamicum* strains transformed with the empty pXMJ19 plasmid (P_*tac*_-empty) or pXMJ19-*pftAB* plasmid (P*_tac_pftAB*, IPTG-inducible) in the presence of 1 mM IPTG. The 95% confidence intervals are shown by the shaded areas. (B) Biomass of the Δ*mctC* P*_tac_pftAB* strain upon entry into stationary phase after growth in MM1+P in the presence of different IPTG concentrations (0, 50, 100, 150, and 1,000 µM). (C) Growth rate of the Δ*mctC* P*_tac_pftAB* strain grown in MM1+P in the presence or absence of IPTG. In panels B and C, mean values ± standard deviations (error bars) from at least six independent experiments are presented. Data were fit with a Michaelis-Menten equation; the 95% confidence intervals of the fits are shown in gray. The data corresponding to the control strains are shown in Fig. S2 in the supplemental material.

We asked whether *pftAB* encodes a pyruvate uptake system. During growth of *C. glutamicum* on pyruvate, pyruvate is taken up by the monocarboxylate transporter MctC, and as a result, a *mctC* mutant is unable to grow on pyruvate as the sole carbon source (16). We therefore tested for functional complementation of the *C. glutamicum mctC* mutant using an IPTG-inducible *pftAB* expression plasmid and appropriate controls. Since the *mctC* mutant cannot grow on pyruvate, we precultured the strains in rich medium, washed the cells, and inoculated a minimal medium plus pyruvate at an optical density of 600 nm (OD_600_) of ~0.02. The expression of *pftAB* was first fully induced using a high IPTG concentration (1 mM). As shown in Fig. 2A, the *mctC* mutant transformed with the empty plasmid was unable to grow on pyruvate as the sole carbon source. Conversely, the strong induction of *pftAB* restored growth of the *mctC* mutant. The biomass reached an OD_600_ of ~0.9 ± 0.1, and the growth rate was ~0.08 ± 0.02 h^−1^ (in comparison with an OD_600_ of ~1 and a growth rate of ~0.16 h^−1^ for the control strain), which indicated that PftAB functionally replaced MctC and enabled pyruvate uptake. In order to check whether the pyruvate uptake, and consequently growth restoration, was limited by the level of expression of PftAB, the experiment was repeated using IPTG at low (50 µM), intermediate (100 and 150 µM), and high (1 mM) concentrations. At low and intermediate levels of *pftAB* induction, the biomass reached an OD_600_ of up to ~0.6 unit (Fig. 2B), and the growth rates were between 0.04 and 0.06 h^−1^ (Fig. 2C and Fig. S2), which indicated that cell growth was IPTG dependent. Altogether, these results indicated that *pftAB* encodes a pyruvate import system.

10.1128/mBio.00976-17.3 FIG S2  Functional complementation of *C. glutamicum* by PftAB. Growth in MM1+P of the WT (diamonds) and Δ*mctC* (squares) *C. glutamicum* strains transformed with the empty pXMJ19 plasmid (filled symbols) or pXMJ19-*pftAB* plasmid (white ovals within symbols). Expression of *pftAB* was induced using different IPTG concentrations (IPTG concentrations indicated by the gray color bar). Mean values ± standard deviations from at least six independent experiments are presented. Download FIG S2, TIF file, 0.1 MB. Copyright © 2017 Charbonnier et al. 2017 Charbonnier et al. This content is distributed under the terms of the Creative Commons Attribution 4.0 International license.

### The PftAB complex is a pyruvate-specific facilitated transporter.

**FIG 3:**
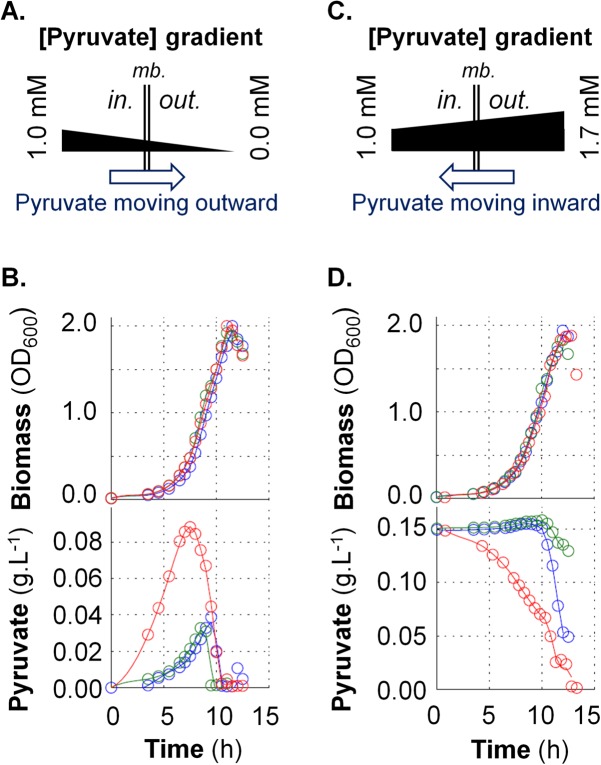
Functional characterization of the PftAB pyruvate transporter in *B. subtilis*. (A and C) Schematic representation of a facilitated transport of pyruvate in the absence (A) or presence (C) of extracellular pyruvate. mb., membrane. (B and D) Growth of the *B. subtilis* WT (blue), Δ*pftAB* (green), and Δ*pftAB* P*_hs_pftAB* (red) strains and corresponding concentrations of extracellular pyruvate. The results from a representative experiment are shown. (B) Cells were grown in M9G with 200 µM IPTG. (D) Cells were grown in M9G with 200 µM IPTG and 0.15 g ⋅ liter^−1^ pyruvate.

We next asked whether PftAB operates as an active or passive pyruvate transport system. A straightforward approach to experimentally address this issue is to evaluate whether PftAB transports pyruvate along or against the concentration gradient of pyruvate. The concentration of intracellular pyruvate in *B. subtilis* cells grown in minimal medium containing glucose is about 1 mM (26). We therefore determined whether PftAB is specifically able to export intracellular pyruvate into minimal medium containing glucose (Fig. 3A). We quantified extracellular pyruvate during growth in M9G (glucose) of the WT, Δ*pftAB*, and P*_hs_pftAB* strains. The three strains exhibited an identical growth phenotype (Fig. 3B, top panel). The extracellular pyruvate measured in the growth medium of the WT and Δ*pftAB* strains steadily increased until the late exponential phase to about 0.03 g ⋅ liter^−1^ (~0.34 mM) with a specific production rate (*q*_Pyr_) of ~0.5 mmol ⋅ h^−1^ ⋅ g of cells^−1^ (dry weight). Afterward, pyruvate concentration dropped to zero, which suggests that cells rapidly used the pyruvate before entering stationary growth phase. This result is consistent with the well-known phenomenon of excretion of pyruvate by *B. subtilis* prior to its (re)assimilation when glucose is depleted (7, 22). With the strain overexpressing *pftAB* (P*_hs_pftAB*), pyruvate culminated at ~0.09 g ⋅ liter^−1^ (~1.02 mM) after 7 h of culture (Fig. 3B, bottom panel). The specific production rate of pyruvate (*q*_Pyr_) peaked at ~5 mmol ⋅ h^−1^ ⋅ g cells^−1^ (dry weight) after 4 h of culture and quickly decreased thereafter. The specific glucose consumption and specific acetate and citrate production rates did not show any differences between the three strains (Fig. S3A to E). These results indicated that PftAB can specifically export pyruvate and also that there exists at least another pyruvate transport system allowing the import and export of pyruvate (consistent with the residual growth on pyruvate of the Δ*pftAB* mutant). Altogether, our findings prompted us to conclude that the gradient of pyruvate drove the PftAB-mediated transport of pyruvate.

10.1128/mBio.00976-17.4FIG S3 Growth of various strains and corresponding extracellular metabolite concentrations. The WT (blue circles), Δ*pftAB* (green circles), and P*_hs_pftAB* (red circles) strains were cultivated on M9G plus 200 µM IPTG (A to E) or M9G plus 0.15 g ⋅ liter^−1^ pyruvate and 200 µM IPTG (F to J). The growth curves for M9G and M9G plus 0.15 g ⋅ liter^−1^ pyruvate are shown in panels A and F, respectively. Extracellular concentrations of pyruvate (maximum specific production rate, $q_{Pyr}^{max}$~ 5 mmol ⋅ h^−1^ ⋅ gDCW^−1^ [gDCW stands for gram dry cellular weight]), citrate ($q_{Cit}^{max}$~ 0.1 mmol ⋅ h^−1^ ⋅ gDCW^−1^), acetate ($q_{Ace}^{max}$~ 5 mmol ⋅ h^−1^ ⋅ gDCW^−1^), and glucose (maximum specific consumption rate, $\nu_{Glc}^{max}$~ 8 mmol ⋅ h^−1^ ⋅ gDCW^−1^) of M9G cultures are shown in panel B, C, D, and E, respectively. Extracellular concentrations of pyruvate ($\nu_{Pyr}^{max}$~ 2 mmol ⋅ h^−1^ ⋅ gDCW^−1^), citrate ($q_{Cit}^{max}$~ 0.l mmol ⋅ h^−1^ ⋅ gDCW^−1^), acetate ($q_{Ace}^{max}$~ 5 mmol ⋅ h^−1^ ⋅ gDCW^−1^), and glucose ($\nu_{Glc}^{max}$~ 8 mmol ⋅ h^−1^ ⋅ gDCW^−1^) of cultures on M9G plus 0.15 g ⋅ liter^−1^ pyruvate are shown in panels B, C, D, and E, respectively. The results of a representative experiment are presented in each panel. Download FIG S3, TIF file, 0.2 MB.Copyright © 2017 Charbonnier et al.2017Charbonnier et al.This content is distributed under the terms of the Creative Commons Attribution 4.0 International license.

To reinforce this conclusion, we cultivated the same strains in M9G supplemented with 0.15 g ⋅ liter^−1^ (~1.70 mM) pyruvate. In this experiment, the pyruvate gradient is reversed so that if PftAB operates as a facilitated transporter, we expect to monitor an import of pyruvate (Fig. 3C). As shown in Fig. 3D, the concentration of extracellular pyruvate measured for the WT strain showed a slight increase until 10 h of culture, followed by a drop as soon as cells entered stationary phase. Interestingly, this drop coincided with the induction of *pftAB* when glucose was depleted (see next paragraphs) (19). Consistently, in the Δ*pftAB* strain, the consumption of pyruvate was delayed and strongly reduced. Conversely, the extracellular concentration of pyruvate measured with the strain overexpressing *pftAB* dropped from the beginning of the culture to reach zero before entry into stationary phase. There were no differences in the specific glucose consumption and specific acetate and citrate production rates in the three strains (Fig. S3F to J). Taken together, these results demonstrated that the gradient of pyruvate drove the PftAB-mediated transport of pyruvate across the cell membrane, which indicated that PftAB operates as a pyruvate-specific facilitated transporter. Making use of a simple model of facilitated transport (Text S1 and Table S2), we estimated that the PftAB complex had a maximum rate (*J*_max_) of approximately 10.0 ± 1.0 mmol ⋅ h^−1^ ⋅ g of cells^−1^ (dry weight) and an apparent affinity constant for pyruvate (*K*_*m*_) of approximately 1.0 ± 0.1 mmol ⋅ liter^−1^.

10.1128/mBio.00976-17.1 TEXT S1  Supplemental Materials and Methods, supplemental Results, and supplemental References. Download TEXT S1, DOCX file, 0.1 MB. Copyright © 2017 Charbonnier et al. 2017 Charbonnier et al. This content is distributed under the terms of the Creative Commons Attribution 4.0 International license.

10.1128/mBio.00976-17.9 TABLE S2  Pyruvate concentrations, specific pyruvate uptake rate and PftAB expression level under various growth conditions. Download TABLE S2, PDF file, 0.01 MB. Copyright © 2017 Charbonnier et al. 2017 Charbonnier et al. This content is distributed under the terms of the Creative Commons Attribution 4.0 International license.

### Pyruvate induces expression of *pftAB* by binding of LytT upstream of the *pftAB* promoter.

**FIG 4:**
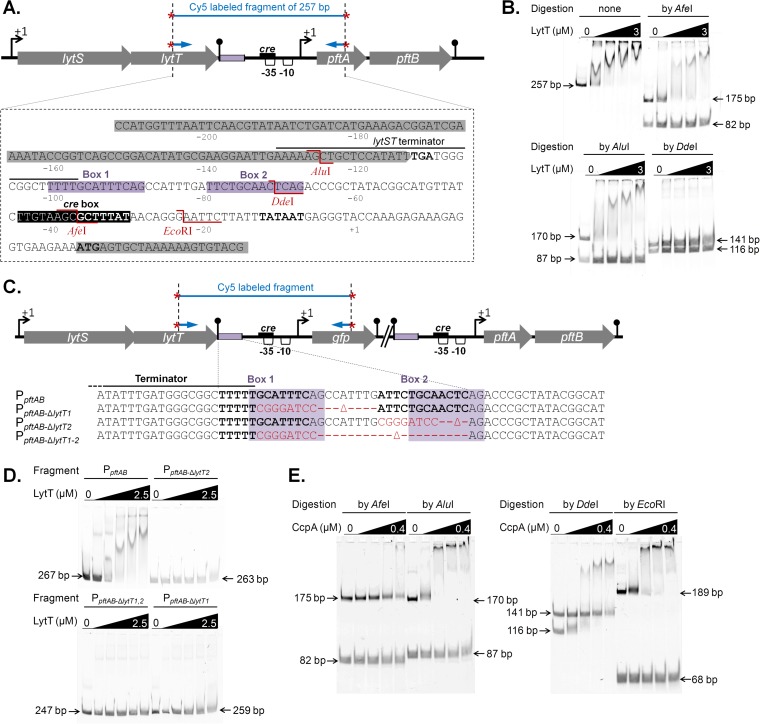
Mapping of the LytT and CcpA binding sites. (A) Genomic organization of the *lytST pftAB* region. At the top of panel A, the black box in the schematic representation indicates the putative binding site of CcpA. The violet boxes indicate the binding region of LytT. The AluI, DdeI, AfeI, and EcoRI restriction sites are indicated in red. (B) EMSAs using purified His_6_-LytT (0, 1, 1.5, 2, and 3 µM) and the Cy5-labeled PCR fragment of 257 bp (represented in panel A) either uncut or digested with AfeI, AluI, or DdeI. (C) The binding region of LytT is detailed for the unmodified (P_*pftAB*_), Box1-deleted (P_*pftAB*-Δ*lytT1*_), Box2-deleted (P_*pftAB*-Δ*lytT2*_), and Box1-Box2-deleted (P_*pftAB*-Δ*lytT1*,*2*_) reporter strains. Black bold letters stand for the two putative LytT DNA binding sequences (Box1, Box2). The red letters indicate the DNA sequence that replaced the deleted region in each strain. The violet shaded regions are similar to the binding sites identified in *E. coli* for the LytT-like YpdA response regulator (35). (D) EMSAs using purified His_6_-LytT (0, 0.5, 1, 1.5, 2, and 2.5 µM) and the Cy5-labeled PCR fragment (represented in panel C) amplified from the P_*pftAB*_ (267 bp), P_*pftAB*-Δ*lytT1*_ (259 bp), P_*pftAB*-Δ*lytT2*_ (263 bp), and P_*pftAB*-Δ*lytT1*,*2*_ (247 bp) reporter strains. (E) EMSAs using purified His_6_-CcpA (0, 0.1, 0.2, 0.3, and 0.4 µM) and P-Ser-HPr (1:10 molar ratio) and the Cy5-labeled PCR fragment of 257 bp (represented in panel A) digested with AfeI (175  and 82 bp), AluI (170  and 87 bp), DdeI (141  and 116 bp), or EcoRI (189 and 68 bp).

The *lytST* operon, which is located immediately upstream of *pftAB*, was recently shown to be involved in the induction of *pftA* upon the entry of Luria-Bertani (LB)-grown *B. subtilis* cells in stationary phase and essential during growth on minimal medium plus pyruvate (19). van den Esker et al. (19) proposed that LytST directly induces *pftAB* based on the chromosomal proximity of these two operons. We tested this assumption *in vitro* by performing electrophoretic mobility shift assay (EMSA) using a DNA fragment of 257 bp containing the intergenic region of the *lytST* and *pftAB* operons (Fig. 4A). As shown in Fig. 4B, increasing amounts of the response regulator His-LytT resulted in a shift in migration of the labeled DNA band, which indicated LytT DNA binding. The possibility of nonspecific binding of LytT was eliminated, as no shifted bands were observed when LytT was incubated with PCR fragments of ~250 bp used as controls (not shown). EMSAs using either AfeI, AluI, or DdeI restriction product of the 257-bp fragment revealed that LytT binds within an 88-bp region bounded by the AfeI and AluI restriction sites and that DdeI cut inside the LytT binding site (Fig. 4B). *In silico* comparative-genomic analysis recently predicted putative binding sites for a series of two-component systems (TCSs) of unknown functions (27). Hence, LytT may bind a DNA sequence within the 88-bp above-mentioned region, which is composed of two boxes of 13 nucleotides separated by 8 nucleotides (Box 1 and Box 2 [Fig. 4A]). We therefore tested this hypothesis using synthetic sequences mutated for these two boxes either independently (P_*pftAB*-Δ*lytT1*_ and P_*pftAB*-Δ*lytT2*_) or together (P_*pftAB*-Δ*lytT1-2*_) (Fig. 4C). LytT shifted the nonmutated DNA fragment but none of the three synthetic fragments (Fig. 4D), which strongly suggested that each of the boxes is required for LytT binding.

In order to *in vivo* validate the LytT binding site, *gfp* transcriptional fusions reporting the promoter activity of *pftAB* using either the wild-type sequence (P*_pftAB_gfp*) or the above-mentioned synthetic sequences (P_*pftAB*-Δ*lytT1*_, P_*pftAB*-Δ*lytT2*_, and P_*pftAB*-Δ*lytT1-2*_) were inserted at the *pftAB* locus in the WT strain and in a Δ*lytST* mutant strain (Fig. 4C and Table 1). As expected, the growth of the Δ*lytST* strain was drastically reduced on pyruvate, while no growth defect was observed when replacing pyruvate by other substrates (Table S1). Because of this growth defect, cells were grown in M9SE with or without pyruvate. The green fluorescent protein (GFP) abundance was ~4.5 units per OD_600_ unit (U ⋅ OD_600_^−^^1^) for WT cells grown in M9SE with pyruvate (M9SE+P) and barely detectable in M9SE (Table 2). In contrast, the GFP abundance was ~0.5 U ⋅ OD_600_^−1^ for the Δ*lytST* strain under both conditions. The GFP abundance from the P_*pftAB*-Δ*lytT1*_, P_*pftAB*-Δ*lytT2*_, and P_*pftAB*-Δ*lytT1-2*_ synthetic reporter strains grown on M9SE+P was similar to that in the Δ*lytST* strain (Table 2). Altogether, these results prompted us to conclude that each of the two predicted boxes is essential for the induction of *pftAB* by the LytST TCS. A genome-wide sequence homology search for other putative LytT binding motifs did not give any significant hits (not shown). Consistently, a comparative analysis performed using two recently published transcriptome data sets, M9P and M9SE (7), revealed that *pftA* and *pftB* were the only genes specifically induced in cells grown in M9P compared to cells grown in M9SE (Fig. S4). Altogether, these results established that pyruvate induced expression of only *pftAB* by binding the LytT response regulator upstream of the *pftAB* promoter.

10.1128/mBio.00976-17.5 FIG S4  Comparative gene expression analysis (gray dots) between cells grown on pyruvate (M9P) versus succinate plus glutamate (M9SE). The *pftA* (red dot) and *pftB* (blue dot) genes are specifically expressed on M9P; the *dctP* (black dot) gene encoding the major C4-dicarboxylate permease is specifically expressed on M9SE. Data were extracted from published data sets (7, 20) and reanalyzed. Download FIG S4, TIF file, 0.1 MB. Copyright © 2017 Charbonnier et al. 2017 Charbonnier et al. This content is distributed under the terms of the Creative Commons Attribution 4.0 International license.

**TABLE 2:** Induction of *pftAB* by the LytST TCS

Strain	Genotype^a^	P_*pftAB*_ expression (U ⋅ OD_600_^−1^)	
		M9SE	M9SE+P
TC35	WT	0.2 ± 0.3	4.4 ± 0.2
TC59	Δ*lytST*	0.7 ± 0.5	0.3 ± 0.4
TC149	P_*pftAB*-Δ*lytT1*_^b^	0.1 ± 0.2	0.3 ± 0.2
TC125	P_*pftAB*-Δ*lytT2*_^b^	0.2 ± 0.3	0.4 ± 0.1
TC126	P_*pftAB*-Δ*lytT1-2*_^b^	0.5 ± 0.7	0.4 ± 0.3

a More-complete relevant genotypes are given in Table 1.

b The deleted LytT binding sites are shown in Fig. 4C.

### Glucose represses transcription of *pftAB* by binding of CcpA to the −35 region of the promoter.

Since PftAB can import and export pyruvate, its expression in cells grown on multiple carbon sources must be tightly regulated to ensure proper pyruvate homeostasis. The promoter of *pftAB* contains a putative *cre* site overlapping the −35 region, which suggests that *pftAB* expression may be under the control of CcpA, the master regulator of carbon catabolite repression (CCR). We tested this hypothesis *in vitro* by conducting EMSAs with increasing amounts of purified CcpA-His, serine-phosphorylated HPr (P-Ser-HPr), and various restriction products of the previously described Cy5-labeled PCR fragment of 257 bp (Fig. 4A). As shown in Fig. 4E, increasing amounts of the CcpA/P-Ser-HPr complex resulted in a shift in migration of fragments of 170 bp for AluI, 116 bp for DdeI, and 189 bp for EcoRI. In contrast, no shift was observed after the fragment was treated by AfeI, which cuts in the putative *cre* box, the binding site for CcpA. These results prompted us to conclude that CcpA binds within a 48-bp region bounded by the DdeI and EcoRI restriction sites.

In order to validate *in vivo* that *pftAB* expression is under the control of the CcpA-dependent catabolite repression, we quantified the P*_pftAB_gfp* expression level in the WT, Δ*ccpA*, *ptsH1*, Δ*crh*, and *ptsH1* Δ*crh* strains under inductive or repressive conditions (Table 3). The deletion of *ccpA* fully relieved the glucose-mediated repression of *pftAB* in M9P with glucose (M9P+G). As expected, the glucose-mediated repression of *pftAB* was maintained in the *ptsH1* and Δ*crh* single mutant strains (Table 3), which confirmed that the two cofactors can functionally replace each other (13). Consistently, the glucose-mediated *pftAB* repression was significantly reduced in the *ptsH1* Δ*crh* double mutant. Hence, glucose repressed transcription of *pftAB* via CcpA and its cofactors by binding of the CcpA/P-Ser-HPr complex or the CcpA/P-Ser-Crh complex to the −35 region of the promoter.

**TABLE 3:** CcpA-dependent and independent catabolite repressions of *pftAB*

Strain	Genotype^a^	P_*pftAB*_ expression (U ⋅ OD_600_^−1^)^b^				
		M9G	M9M	M9P	M9P+G	M9P+M
TC35	WT	<0.1	0.6 ± 0.1	4.5 ± 0.2	0.6 ± 0.2	0.4 ± 0.2
TC36	Δ*ccpA*	<0.1	0.3 ± 0.2	4.8 ± 0.2	3.5 ± 0.5	0.4 ± 0.1
TC101	*ptsH1*	<0.1	0.5 ± 0.2	4.9 ± 0.6	<0.1	0.9 ± 0.2
TC100	Δ*crh*	0.1 ± 0.1	0.4 ± 0.1	5.3 ± 0.6	<0.1	1.0 ± 0.2
TC102	*ptsH1* Δ*crh*	<0.1	0.2 ± 0.1	4.8 ± 0.5	2.8 ± 0.2	0.5 ± 0.1
TC90	Δ*ccpA* Δ*maeA*	*–*	*–*	4.9 ± 0.2	*–*	4.8 ± 0.5
TC91	Δ*ccpA* Δ*ywkB*	*–*	*–*	4.9 ± 0.6	*–*	0.6 ± 0.1
TC105	Δ*ccpA* Δ*maeA* P*_hs_malS*	*–*	*–*	4.4 ± 0.4	*–*	3.7 ± 0.3
TC105 + I^c^	Δ*ccpA* Δ*maeA* P*_hs_malS* + I^c^	*–*	*–*	5.2 ± 0.4	*–*	0.4 ± 0.2

a More-complete genotypes are given in Table 1.

b –, not determined.

c I, inducer. Overexpression of *malS* was carried out using 200 µM IPTG.

### Malate represses transcription of *pftAB* by a malic enzyme-dependent but CcpA-independent mechanism.

Malate was recently shown to hijack the usual CcpA-mediated catabolite repression (13). Consistently, the expression level of P*_pftAB_gfp* was ~0.4 U ⋅ OD_600_^−1^ for WT cells grown in M9P with malate (M9P+M), which is about 9% of the expression level in M9P (Table 3). However, the malate-mediated repression of *pftAB* was not relieved in the Δ*ccpA* mutant or in any of the Δ*ccpA*, *ptsH1*, Δ*crh*, and *ptsH1* Δ*crh* mutants (M9P+M medium [Table 3]). These results prompted us to conclude that malate repressed *pftAB* transcription by at least one CcpA-independent mechanism.

To identify the key players of this CcpA-independent mechanism, a mini-Tn*10* insertion library was constructed from a *B. subtilis pftAB* reporter strain in a Δ*ccpA* background. This library was then screened on plates for *pftAB* derepression in the presence of malate. Thirteen positive clones from six independent pools of transposants were isolated and further characterized (Fig. S5). Most of the clones with strong *pftAB* derepression were mutated in the TCS *malK*/*malR* and its regulon involved in malate transport (*maeN*) and utilization (*maeA*) (6, 28). As *maeA* is the first gene of the *maeA ywkB* operon, a possible role of *ywkB* (of unknown function) could not be excluded. We thus analyzed the effect of the deletion of each gene. The inactivation of *maeA* fully relieved *pftAB* expression in M9P+M, while the inactivation of *ywkB* had no significant effect (Table 3). These results showed that the CcpA-independent malate-dependent repression requires *maeA*.

10.1128/mBio.00976-17.6 FIG S5  Transposon mutagenesis identified genes involved in the malate-induced CcpA-independent catabolite repression of *pftAB*. Genes were identified by random mutagenesis by transposition (~14,000 clones screened). The insertion sites of the mini-Tn*10* transposon in the *B. subtilis* genome are indicated as follows. The light gray triangles indicate an insertion in one clone, and the dark gray triangles indicate one insertion in two independent clones. The *malK*, *malR*, *maeN*, and *maeA* genes are involved in the malate assimilation pathway with the *malKR* operon encoding a two-component system, *maeN* encoding the principal malate transporter, and *maeA* encoding a malic enzyme. The expression of *maeN* and *maeA* is under the control of the MalKR two-component system, and the binding sites of MalR are indicated as light gray boxes upstream of *maeA* and *maeN*. The *srfAA* and *bioW* genes are involved in surfactin production and biotin synthesis, respectively. Download FIG S5, TIF file, 0.04 MB. Copyright © 2017 Charbonnier et al. 2017 Charbonnier et al. This content is distributed under the terms of the Creative Commons Attribution 4.0 International license.

*B. subtilis* possesses four paralogous malic enzymes: YtsJ, a NADP-dependent malate dehydrogenase, plays the major role in malate utilization, whereas MaeA, MalS, and MleA are NAD dependent and are dispensable for growth on malate (29). The CcpA-independent repression of *pftAB* was dependent on the NAD-dependent activity, as the overexpression of *malS* in a Δ*maeA* strain fully restored the CcpA-independent catabolite repression (Table 3). Altogether, our results indicated that the NAD-dependent malic enzyme activity but not the MaeA protein *per se* is essential to drive the malate-mediated CcpA-independent repression of *pftAB*.

### The higher the pyruvate influx and/or the intracellular pyruvate concentration, the stronger the CcpA-independent repression of *pftAB*.

**FIG 5:**
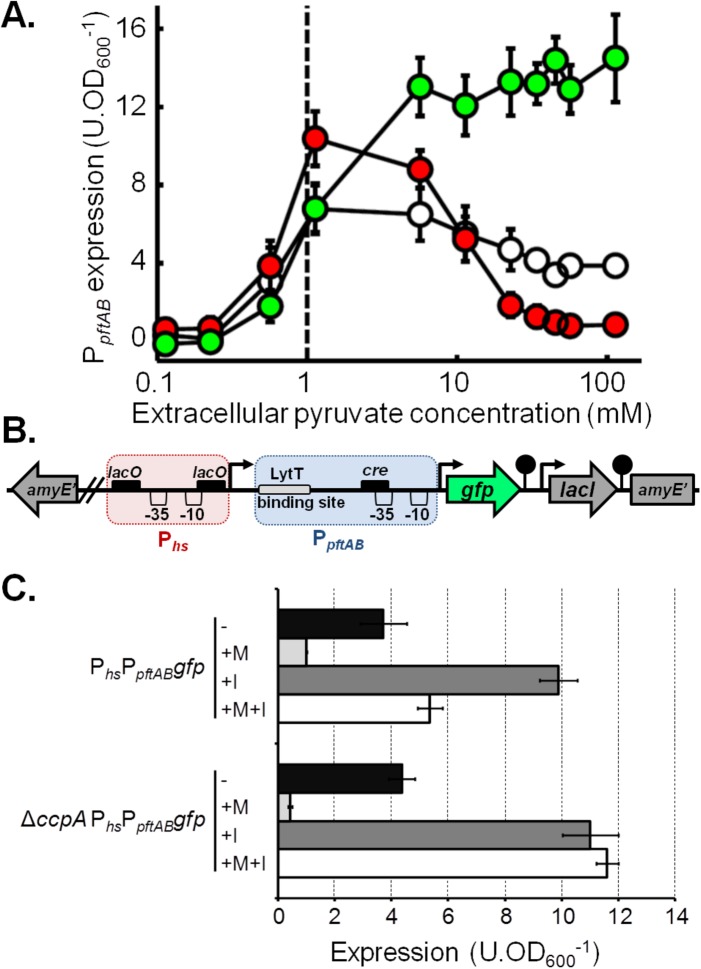
Pyruvate influx tightly controls *pftAB* expression. (A) Expression of *pftAB* in the WT (white), Δ*pftAB* (green), and P*_hs_pftAB* (red) strains grown in M9SE+P (at pyruvate concentrations ranging from 0.1 to 100 mM). The P*_hs_pftAB* strain was grown with 1 mM IPTG. The dashed line represents the estimated *K*_*m*_ of PftAB. (B) Genomic structure of the P_*hs*_P_*pftAB*_*gfp* at the *amyE* locus. The red and blue boxes represent P_*hs*_ and P_*pftAB*_, respectively. Black boxes indicate DNA binding sites: *cre* for CcpA and *lacO* for LacI. (C) Expression of P_*hs*_P_*pftAB*_*gfp* in the WT and Δ*ccpA* strains grown in M9SE+P in the presence (+) or absence (−) of malate (M) or 200 μM IPTG (I). Expression was estimated in the exponential phase of growth; mean values ± standard deviations from at least six experiments are presented in panels A and C.

The NAD-dependent malic enzyme MaeA catalyzes the transformation of malate into pyruvate during *B. subtilis* growth on malate (7). This prompted us to hypothesize that the end product of the reaction (i.e., intracellular pyruvate) is responsible for the CcpA-independent repression of *pftAB*. To test this hypothesis, we manipulated the concentration of intracellular pyruvate by two complementary means, making use of the kinetic properties of the PftAB transport system. P*_pftAB_gfp* reporter cells were grown in M9SE supplemented with different concentrations of pyruvate, and the rate of pyruvate uptake was controlled by modulating the level of PftAB (using the WT, Δ*pftAB*, and P*_hs_pftAB* strains) (Fig. 5A). Hence, if the intracellular pyruvate negatively affects *pftAB* expression, we will monitor in the presence of extracellular pyruvate a lower P_*pftAB*_ activity in the WT and IPTG-induced P*_hs_pftAB* strains than in the Δ*pftAB* mutant, which is unable to take up pyruvate. GFP abundances in the WT and Δ*pftAB* strains were similar for extracellular pyruvate concentrations below 1 mM (note that this is the estimated *K*_*m*_ of PftAB). For higher extracellular pyruvate concentrations, GFP abundance increased up to 14 U ⋅ OD_600_^−1^ in the Δ*pftAB* strain, while it slowly decreased down to 4 U ⋅ OD_600_^−1^ for the WT strain. Remarkably, GFP abundance in the IPTG-induced P*_hs_pftAB* strain peaked at ~11 U ⋅ OD_600_^−1^ for an extracellular pyruvate concentration of about 1 mM and dropped afterward down to ~1 U ⋅ OD_600_^−1^ (Fig. 5A). These results indicated that, although extracellular pyruvate triggered the induction of *pftAB*, this induction is strongly inhibited (14-fold between the Δ*pftAB* and P*_hs_pftAB* strains) by the activity of PftAB, i.e., by pyruvate uptake. It is worth noting that this feedback regulation led to a repression of *pftAB* of ~90%, which is similar to the level of the malate-dependent, CcpA-independent repression. Hence, the malate-dependent, CcpA-independent repression of *pftAB* most probably results from the strong increase in pyruvate influx and/or concentration generated by the high malic enzyme activity due to the induction of *maeA* in the presence of malate.

### The elevated pyruvate influx and/or concentration seems to alter the induction of *pftAB* by LytST.

Although no DNA-binding regulatory protein other than MalR was identified by our transposon mutagenesis, the high malic enzyme flux in the presence of malate may activate a yet unidentified regulator responsible for the CcpA-independent repression of *pftAB*. Alternatively, the CcpA-independent repression of *pftAB* may be mediated by the LytST TCS itself, i.e., by reducing its activator activity. In order to test these assumptions, we constructed a synthetic fusion of the IPTG-inducible P_*hs*_ and the native P_*pftAB*_ upstream of the *gfp* gene (P_*hs*_P_*pftAB*_*gfp*) (Fig. 5B). If the CcpA-independent repression of *pftAB* is mediated by an unknown DNA-binding repressor acting on or downstream of P_*pftAB*_, the expression driven by the upstream P_*hs*_ will be altered by this repressor, somewhat acting as an artificial roadblock. In contrast, if the CcpA-independent repression of *pftAB* results from a lower activation of LytST, the expression from P_*hs*_ will be fully relieved in the presence of IPTG. As shown in Fig. 5C, the malate-dependent repression was maintained on the synthetic P_*hs*_P_*pftAB*_*gfp* with or without IPTG. However, in the Δ*ccpA* mutant, while the CcpA-independent repression by malate was maintained in the absence of IPTG, the repression was fully relieved in the presence of IPTG (Fig. 5C). This experiment revealed that CcpA repressed expression from the synthetic P_*hs*_P_*pftAB*_*gfp* in the presence of malate by binding on the −35 region of P_*pftAB*_ (as in the presence of glucose) and by acting as a roadblock for the transcribing RNA polymerase recruited by P_*hs*_ (Fig. 5B). Altogether, these results suggested that there is no other DNA-binding protein that repressed P_*pftAB*_ in the presence of malate, which implies that the CcpA-independent *pftAB* repression actually resulted from a lower level of induction of *pftAB* by LytST.

## DISCUSSION

**FIG 6:**
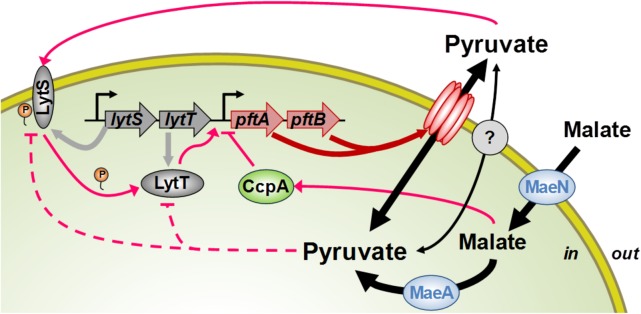
Roles of PftAB and LytST in pyruvate homeostasis. The products of the *pftAB* operon form a hetero-oligomeric membrane complex encoding the major pyruvate import/export system in *B. subtilis*. The LytST TCS senses the extracellular pyruvate concentration and responds by inducing *pftAB* transcription. The accumulation of intracellular pyruvate (or of an intermediate of overflow metabolism) reduces the level of induction of *pftAB* via LytST. This accumulation results either from the uptake and metabolism of pyruvate or from the uptake of malate (by MaeN) and its consecutive transformation into pyruvate by the malic enzyme MaeA. Malate (and glucose) also triggers the catabolite repression of *pftAB* via CcpA. There is at least one other pyruvate transporter yet to be identified (gray filled circle). P, phosphate.

In this study, we characterized the molecular and physiological logics of the pyruvate-induced response of a novel pyruvate transporter in *B. subtilis*. This novel bacterial transport system consists of a hetero-oligomeric complex of PftA and PftB which operates as a pyruvate-specific facilitated transporter. In the presence of extracellular pyruvate, the *pftAB* operon is induced by the TCS LytST. As for the transporters of alternative substrates, *pftAB* expression is repressed by the CcpA-dependent catabolite repression when a preferred carbon source, glucose or malate, is present in the medium. Unexpectedly, however, in the absence of preferred carbon source when the pyruvate influx is high, LytST activity is drastically retroinhibited (Fig. 6). Hence, LytST constitutes, together with the transporter PftAB, an original regulatory system ensuring proper adaptation to changing environments.

It was recently shown in *B. subtilis* forming biofilms that *pftAB* is induced in the presence of extracellular acetate (30). When we added acetate to the medium, we observed no induction of *pftAB* in exponentially growing cells. However, we observed a weak, dose-dependent induction when cells reached the stationary phase after growth in repressive conditions (data not shown). A reasonable hypothesis is that the reduction of the acetate export consequent to the presence of extracellular acetate led to an overflow metabolic shift toward an increase of pyruvate export. Indeed, it is well-known that when the intracellular pyruvate pool is high, the overflow metabolism, in particular pyruvate export, is stimulated (7). We showed that pyruvate export during exponential growth on glucose is independent from PftAB which is kept under the control of the catabolite repression (Fig. 6). Hence, at the onset of the stationary phase when the CcpA-dependent repression is relieved, the exported pyruvate triggered *pftAB* induction in a dose-dependent manner.

The *pftA* and *pftB* genes were originally annotated as encoding homologs of the *Staphylococcus aureus* LrgA and LrgB membrane proteins (18). The molecular mechanisms controlling death and lysis during biofilm development of *S. aureus* are organized around the CidR-regulated *cidABC* and LytSR-regulated *lrgAB* operons. *cidA* and *lrgA* encode proteins that are believed to function as holin and antiholin, respectively, while *cidB* and *lrgB* are of unknown function (31). Although PftA and PftB share strong homologies with holin/antiholin systems, the finding that these proteins belong to a new class of effective bacterial transporters is consistent with the recent discovery of the chloroplastidic glycolate/glycerate transporter, PLGG1, that most likely evolved from a gene fusion of bacterial *lrgA* (*pftA*) and *lrgB* (*pftB*) homologs (32, 33). Also consistent with our findings in *B. subtilis*, an impaired ability to utilize pyruvate was observed in the *lytST*-like *lytSR* mutant of *Staphylococcus epidermidis* (34). In addition, it was recently shown in the evolutionarily distant bacterium *Escherichia coli* that the LytST-like YpdAB TCS is weakly activated by pyruvate (35, 36). Remarkably, the YpdB binding site is similar to the *B. subtilis* LytT binding site (Fig. 4C). However, it was proposed that in *S. aureus*, LytSR senses decrease in membrane potential and responds by inducing *lrgAB* transcription. It was also proposed that a primary intermediate of overflow metabolism, acetyl phosphate (acetyl-P), already shown to act as a small phosphodonor to response regulators (37), directly activates LytR in an alternative signaling pathway (31). Our data, however, demonstrated that the higher the pyruvate influx, the lower the induction level of *pftAB* (Fig. 5A). This finding argues in favor of a negative-feedback regulation by the level of intracellular pyruvate (Fig. 6). Besides, we observed no induction of *pftAB* in either the WT or Δ*ccpA* strain under conditions that are known to give rise to elevated concentrations of the intermediates of overflow metabolism and high acetate excretion rates (7, 22). Hence, if acetyl-P acts as a phosphate donor to LytT in *B. subtilis*, it does not result in its activation, as has been proposed for *S. aureus* LytR. Overall, there are undeniable similarities in gene sequences and signaling between the homologous staphylococcal LytSR, *E. coli* YpdAB, and *B. subtilis* LytST TCSs but also significant physiological and functional divergences, which are likely to be related to niche-specific evolutionary constraints.

Unpredictable changing environments necessitate appropriate responses for successful bacterial adaptation. Appropriate growth strategies rely on sensing systems that globally adjust gene expression via transcription factor-mediated feed-forward and feedback regulations. In particular, bacterial TCSs combine a sensor (i.e., the sensor kinase) with a feed-forward controller (i.e., the response regulator) to induce expression of target genes involved in the adaptation process. The *pftAB* expression levels in response to different pyruvate concentrations (Fig. 5A) revealed the existence of an additional feedback control acting on LytST. To gain insight into the molecular mechanism that governed the opposed, feed-forward and feedback LytST-mediated regulation of *pftAB* by extracellular and intracellular pyruvate, we developed a simple model of gene expression and explored competitive, noncompetitive, and uncompetitive inhibitions of LytT activation by intracellular pyruvate (see Text S1 in the supplemental material). We assumed that the LytT regulatory protein binds to the *pftAB* promoter as a homodimer upon phosphorylation by LytS, which can be well represented by a Hill equation (38), but phosphorylation of LytT by LytS has not yet been demonstrated. The model simulations perfectly mimicked the induction of the *pftAB* promoter in the Δ*pftAB* strain (Fig. S6). However, the model could not account for *pftAB* induction in the other genetic backgrounds, in which cells are still capable of utilizing extracellular pyruvate (Text S1). Other molecular mechanisms may explain the feedback regulation of LytST by intracellular pyruvate, such as inhibition of LytS autophosphorylation or hindered recruitment of the RNA polymerase (Fig. 6) or a phosphorylation state-dependent proteolysis of LytT. To decide between these mechanisms and verify to which extent they apply to other TCSs, characterization of the transduction signal and of the *pftAB* regulation dependency on the LytT phosphorylation state should be performed. Indeed, such a retroinhibition challenges the rational design of novel nature-inspired sensors and synthetic switches but undoubtedly offers new possibilities for the development of integrated sensor/controller circuitry.

10.1128/mBio.00976-17.7 FIG S6  Intracellular pyruvate and pyruvate influx tightly control the expression of *pftAB*. Expression of *pftAB* in the WT (white circles), Δ*pftAB* (green circles), and P*_hs_pftAB*(grown with 1 mM IPTG) (red circles) strains grown in M9SE plus pyruvate (at concentrations ranging from ~0.1 to ~100 mM) are shown on a linear and log scale *x* axis in panels A and B, respectively. Data points correspond to data from about 350 independent cultures, and the mean values are shown in Fig. 5. Fitting the expression data set of the Δ*pftAB* mutant according to a Hill equation (black plain line) (Text S1) revealed a maximal activity of ~7,000 s^−1^ and a Hill coefficient of 2. Download FIG S6, TIF file, 0.1 MB. Copyright © 2017 Charbonnier et al. 2017 Charbonnier et al. This content is distributed under the terms of the Creative Commons Attribution 4.0 International license.

In conclusion, the influx and efflux of pyruvate from exponential to stationary growth phases result from the timely (re)routing of the central carbon metabolic fluxes and of the dynamics of the intermediate concentrations. Hence, the intracellular and extracellular sensor systems and the feed-forward and feedback regulations of pyruvate uptake ensured with respect to cell adaptation a tight management of pyruvate homeostasis.

## MATERIALS AND METHODS

### Media and bacterial strains.

*Escherichia coli* DH5α and TG1 were used for plasmid construction and transformation using standard techniques (39). *Bacillus subtilis* and *Corynebacterium glutamicum* strains used in this study were verified by sequencing and are listed in Table 1. *B. subtilis* strains were derived from BSB168, a *trp*^+^ derivative of *B. subtilis* 168 (20, 22). Luria-Bertani (LB) broth was used to grow *E. coli*, *B. subtilis*, and *C. glutamicum* for transformation procedures only. For other experiments, *B. subtilis* was grown in a modified M9 medium (40) and when necessary supplemented with 25 mg ⋅ liter^−1^ isoleucine, 50 mg ⋅ liter^−1^ leucine, 40 mg ⋅ liter^−1^ valine, 20 mg ⋅ liter^−1^ methionine, and 4 g ⋅ liter^−1^ glutamate (41). *C. glutamicum* was grown in MM1 minimal medium (16). Carbon sources were used at concentrations of 3 g ⋅ liter^−1^ glucose, 5 g ⋅ liter^−1^ malate, 4 g ⋅ liter^−1^ succinate plus 4 g ⋅ liter^−1^ glutamate, or 6 g ⋅ liter^−1^ pyruvate. When required, media were supplemented with antibiotics at the indicated concentrations: for *E. coli*, ampicillin (100 μg ⋅ ml^−1^), spectinomycin (200 μg ⋅ ml^1^); for *B. subtilis*, spectinomycin (100 µg ⋅ ml^−1^), kanamycin (5 µg ⋅ ml^−1^), erythromycin (1 µg ⋅ ml^−1^), phleomycin (1 µg ⋅ ml^−1^), chloramphenicol (5 µg ⋅ ml^−1^); for *C. glutamicum*, chloramphenicol (30 µg ⋅ ml^−1^).

### Gene deletion.

Deletions of *pftA* (*ysbA*), *pftB* (*ysbB*), *pftAB* (*ysbAB*), and *lytST* were performed by sequence replacement with a chloramphenicol resistance cassette (Cm^r^) expressed under the control of a constitutive promoter. The Cm^r^ cassette was PCR amplified from plasmid pDG1661 using primers containing either a ClaI, SmaI, or XbaI restriction site (see Table S3 in the supplemental material). PCR products were purified using the Wizard SV gel and PCR clean-up system (Promega, Madison, WI) and then digested by the proper restriction enzymes. Regions containing *pftA*, *pftB*, *pftAB*, and *lytST* were PCR amplified from genomic DNA using primers listed in Table S3. PCR products were purified and subcloned into the pGEM-T Easy vector according to the supplier’s protocol. Reverse PCR were performed on the resulting plasmids using primers containing either the ClaI, SmaI, or XbaI restriction site (Table S3). After purification and digestion, PCR products were ligated to the corresponding Cm^r^-containing PCR product.

10.1128/mBio.00976-17.10 TABLE S3  Relationships between *B. subtilis* and *C. glutamicum* strains, plasmids, and primers used in this work. Download TABLE S3, PDF file, 0.01 MB. Copyright © 2017 Charbonnier et al. 2017 Charbonnier et al. This content is distributed under the terms of the Creative Commons Attribution 4.0 International license.

### Inducible gene expression.

Isopropyl-β-d-thiogalactopyranoside (IPTG)-inducible expression of *lytST* was performed by fusing *lytST* to the P_*spac*_ promoter from pMUTIN4 using BamHI and SacI restriction sites, followed by the single crossover integration of the resulting plasmid upstream *pftAB* in the Δ*lytST* strain. IPTG-inducible expression of *malS* and *pftAB* were carried out by fusing these genes to the P_*hs*_ promoter using plasmids pDR111 (kind gift of David Rudner), pDG1661, and pDG1664 (42). Briefly, PCR-amplified fragments (Table S3) of *pftAB* and *malS* were cloned into the pDR111 plasmid by SalI/NheI digestion leading to pDR111-P*_hs_pftAB* and by SalI/SphI digestion leading to pDR111-P*_hs_malS*. A PCR on pDG1664 was performed to introduce a NotI restriction site using appropriate primers (Table S3). The P*_hs_pftAB* from pDR111-P*_hs_pftAB* was then cloned into pDG1664-*Not*I using BamHI and NotI restriction sites. The spectinomycin resistance cassette (Spec^r^) of pDR111-P*_hs_malS* was replaced by Cm^r^ from pDG1661 using EcoRI and SacI. The pDR111, pDG1661, and pDG1664 derivative plasmids were inserted by double crossover at the *amyE* and *thrB* loci, respectively. IPTG-inducible expression of *pftAB* for complementation of the *C. glutamicum mctC* mutant was performed by inserting the PCR-amplified sequence of *pftAB* (Table S3) in the pXmj19 plasmid (kind gift of Gerd Seibold) using SmaI and SalI restriction sites.

### Promoter reporter fusions.

Fusion of the P_*pftAB*_ promoter with *lacZ* (for transposon mutagenesis) was constructed using the pDG1661 plasmid and the PCR-amplified P_*pftAB*_ using appropriate primers (Table S3). The PCR fragment was inserted by HindIII/BamHI restriction/ligation. Fusion of P_*pftAB*_ with the *gfpmut3* gene was carried out by ligation-independent cloning using the pBSBII plasmid (Table S3) as described previously (43). Reverse PCR on the pBSBII-P_*pftAB*_ plasmid using proper primers (Table S3) was used to substitute parts or all of the LytT binding site from the promoter region of *pftAB* by BamHI. The pBSBII derivative plasmids were inserted at the *pftAB* locus by single crossover. Fusion of the P_*hs*_ and P_*pftAB*_ promoters upstream of *gfpmut3* was performed by PCR amplification of the P_*pftAB*_*gfp*-containing region from the pBSBII-P_*pftAB*_ using appropriate primers (Table S3) and subsequent insertion downstream of P_*hs*_ into pDR111. The resulting plasmid was integrated by double crossover at the *amyE* locus, which led to the P_*hs*_P_*pftAB*_*gfp* transcriptional fusion.

### Live-cell array and fluorescence analysis.

Experiments and analyses were performed as previously described (22, 43). Cells were grown in 100 µl of medium in 96-well plates (Cellstar; Greiner Bio-One) and incubated at 37°C under constant shaking in a Synergy II microplate reader (BioTek). The optical density at 600 nm (OD_600_) and fluorescence were measured every 10 min. Each culture was performed in at least three technical replicates by two biological replicates (more than six values). The mean green fluorescent protein (GFP) concentration in exponentially growing cells was expressed as unit per OD_600_, with 1 unit being equivalent to 1 pM fluorescein (Fig. S6).

### Transposon mutagenesis.

The mini-Tn*10* delivery vector pIC333 was used for transposon mutagenesis as previously described (44, 45). This plasmid was introduced into *B*. *subtilis* strain TC64 (Table 1) at 25°C using erythromycin selection. Single transformants were used to inoculate independent cultures at 25°C in LB plus spectinomycin. In early exponential growth, the temperature was shifted to 40°C, and cultures were allowed to grow for 4 more hours. Appropriate culture dilutions were spread on solid M9 medium with pyruvate and malate (M9P+M) plus spectinomycin and 0.04% X-Gal (5-bromo-4-chloro-indolyl-β-d-galactopyranoside) and incubated at 25°C to screen for clones with derepressed P*_pftAB_lacZ* expression (blue colonies). Thirteen clones from independent pools of transposants were isolated in which the *lacZ* gene was expressed on M9P+M (Fig. S4). These clones were selected for backcross experiments, and genes inactivated by the transposon were identified by target rescue and sequencing.

### PftA and PftB localization and quantitative pulldown assays.

Fusion of the SPA (sequential peptide affinity) sequence at the 3′ end of either *pftA* or *pftB* was performed using the pMUTIN-LICSPA plasmid, previously adapted from the pMUTIN-SPA plasmid for ligation-independent cloning to facilitate high-throughput (HTP) cloning (46) and appropriate primers (Table S3). Each SPA-tagged strain was precultured overnight in the presence of erythromycin at 37°C. The culture was initiated by a 500-fold dilution (to an OD_600_ of 0.001) in 2 liters of M9 medium with glutamate and succinate plus pyruvate (M9SE+P). When the OD_600_ reached 0.3, cultures were centrifuged at 3,000 × *g* for 10 min at 4°C. The pellet was resuspended in 50 ml of cold buffer (10 mM Tris-Cl [pH 8], 150 mM NaCl) and instantly frozen in liquid nitrogen. The cells were disrupted using a French press. Membrane and cytosolic fractions were separated by ultracentrifugation at 100,000 × *g* for 1 h. The membrane pellet was solubilized using buffer A (10 mM Tris-Cl [pH 7.5], 150 mM NaCl, 1 mM EDTA) supplemented with 1% DDM (*n*-dodecyl-d-maltoside). Protein concentrations were measured by the Bradford method, and identical protein amounts of the two fractions were loaded on 10% SDS-polyacrylamide gels for Western blotting using anti-FLAG antibodies. In addition, protein complexes from the membrane fraction were pulled down using a SPA purification method (47), then separated by native polyacrylamide gel electrophoresis (PAGE), and analyzed by nanoscale liquid chromatography coupled to tandem mass spectrometry (nanoLC-MS/MS) (Text S1).

### Affinity capture of the SPA-PftA PftB-His complex.

The *pftAB* operon was PCR amplified from the *B*. *subtilis* BSB168 chromosome with primers OOD141 and OOD142 (Table S3). The OOD142 primer allowed the PftB C-terminal fusion of six His residues. After digestion by XhoI and NotI, the PCR fragment was ligated to the pSG-SPA-NTER plasmid (48), enabling the PftA N-terminal fusion of SPA and placing the *SPA-pftA pftB-His*_*6*_ synthetic operon under the control of a xylose-dependent promoter P_*xyl*_. The plasmid was used to generate strain TC199 by double crossover at the *amyE* locus. Transformants were selected for resistance to spectinomycin and lack of amylase activity. Expression of *SPA-pftA pftB-His* was induced by 1% xylose (vol/vol) at mid-log growth and harvested 3 h later. Cell membranes were prepared after cell disruption by sonication in buffer A and centrifugation at 100,000 × *g* for 1 h. Membranes were solubilized using buffer A supplemented with 1% *n*-dodecyl-β-d-maltoside (DDM). The solution was either mixed with nickel-nitriloacetic acid (Ni-NTA) agarose resin (Invitrogen) after the addition of 10 mM imidazole or mixed with the CaM Sepharose-4B resin (GE Healthcare) after the addition of 2 mM CaCl_2_ and left overnight at 4°C on a rotating wheel. For the Ni-NTA agarose column, washes were performed with buffer A supplemented with 20 mM imidazole, and the elution was performed with 250 mM imidazole. For the CaM Sepharose-4B column, washes were performed by buffer A supplemented with 0.1 mM CaCl_2_, and the elution was performed with buffer A supplemented with 3 mM EGTA. Both columns were also used sequentially, in both ways. Prior to the analyses by Western blotting, proteins were precipitated by the addition of 10% trichloroacetic acid (TCA).

### Electrophoretic mobility shift assay.

The *lytT* coding sequence was PCR amplified using primers enabling insertion of a His_6_ tag between the start codon and the coding sequence and cloning into the *E. coli* pJ411 expression vector (DNA2.0, Newark, CA), and His_6_-LytT proteins were expressed in strain ER2566 (NEB). The His_6_-CcpA protein was expressed from a pQE30 derivative vector (kind gift of Anne Galinier). Cells were grown at 30°C in 1 liter LB medium supplemented with the required antibiotics, and expression was induced for 3 h by the addition of 500 µM IPTG when biomass reached an OD_600_ of 0.7. The cells were harvested by centrifugation, resuspended in 40 ml of 50 mM Tris-HCl (pH 8.0) and 1 M NaCl, sonicated, and centrifuged at 100,000 × *g* for 1 h at 4°C. Supernatants were loaded onto a preequilibrated Ni^2+^ affinity column (Ni-NTA agarose; Qiagen), and the His_6_-tagged proteins were purified by successive washing steps in 50 mM Tris-HCl (pH 8.0) and 1 M NaCl with increasing concentrations of imidazole (0 to 20 mM; Text S1). Samples eluted with 50 mM Tris-HCl (pH 8.0), 1 M NaCl, and 250 mM imidazole were dialyzed against a solution containing 50 mM Tris-HCl (pH 8.0), 0.4 M NaCl, 50% glycerol, 1 mM dithiothreitol (DTT). The dialyzed fraction containing the His_6_-LytT protein was further loaded onto a 1 ml Hitrap heparin column (GE) to remove contaminants (Text S1). The P_*pftAB*_ DNA substrate for His_6_-CcpA and His_6_-LytT was PCR amplified from strain BSB168 using Cy5-labeled primers (P1, CCATGGTTTAATTCAACGTATAATC; P2, CGTACACTTTTTTAGCACTCATTTTCTTCACC) while the P_*pftAB*-Δ*lytT1*_, P_*pftAB*-Δ*lytT2*_, and P_*pftAB*-Δ*lytT1-2*_ substrates were amplified from strains TC149, TC125, and TC126, respectively, using primer P1 and an unlabeled primer (P3, CGTACACTTTTTTAGCACTCATTTTCTTCACC). Protein-DNA interactions were evaluated by electrophoretic mobility shift assays (EMSAs). For EMSAs with His_6_-LytT, 50 ng of DNA substrate was used with 50 µg ⋅ ml^−1^ of nonspecific competitor poly(dI-dC). For EMSAs with His_6_-CcpA, the His_6_-CcpA protein was mixed with the serine-phosphorylated HPr (kind gift of Josef Deutscher) in a 1:10 molar ratio, and 10 ng of DNA substrate was used with 400 µg ⋅ ml^−1^ sheared salmon sperm DNA competitor. Samples were loaded onto a 6% or 8% acrylamide (19:1) native gel in Tris-acetate-EDTA (TAE), migration was carried out at 20 V ⋅ cm^−1^ and imaged on a ChemiDoc imaging system (Bio-Rad).

### Physiological parameters.

Extracellular substrate and by-product concentrations were measured by high-performance liquid chromatography (HPLC) using a Dionex UltiMate 3000 rapid-separation liquid chromatography (RSLC) system (Thermo Fisher Scientific, Waltham, MA, USA). Sugars were detected with a refractive index detector, and organic acids were detected with a UV-visible (UV-Vis) detector. Substrate or product yields were calculated by linear regression of external concentration against biomass, and specific rates were calculated as yield multiplied by the growth rate. At least 10 time points during the exponential growth phase were used for the regression analysis.
