# Supplementary material for: Molecular and Physiological Logics of the Pyruvate-Induced Response of a Novel Transporter in Bacillus subtilis
Source: mBio. 2017 Oct 3;8(5):e00976-17. doi: 10.1128/mBio.00976-17 (PMC5626966; doi:10.1128/mBio.00976-17)
Supplement: TABLE S1 [file mbo005173508st1.pdf]

Table S1. Growth rates of wild-type, mutants and complemented strains in M9 plus various substrates

Strains	Growth rate (µ, h^{-1})			
	M9P	M9G	M9M	M9SE
BSB168	0.25 ± 0.08	0.74 ± 0.04	0.83 ± 0.05	0.52 ± 0.08
TC58	-[b]	0.67 ± 0.03	0.80 ± 0.04	0.52 ± 0.08
TC60	-[b]	0.70 ± 0.03	0.80 ± 0.05	0.49 ± 0.04
TC61	-[b]	0.71 ± 0.01	0.78 ± 0.04	0.52 ± 0.05
TC62	-[b]	0.72 ± 0.03	0.79 ± 0.04	0.53 ± 0.05
TC73[a]	0.28 ± 0.07	0.67 ± 0.02	0.78 ± 0.03	0.56 ± 0.03
TC74[a]	0.25 ± 0.05	0.66 ± 0.02	0.81 ± 0.02	0.47 ± 0.06
TC75[a]	0.26 ± 0.04	0.62 ± 0.07	0.76 ± 0.05	0.45 ± 0.06
TC76[a]	0.25 ± 0.05	0.65 ± 0.06	0.76 ± 0.04	0.47 ± 0.08

[a] the growth medium was supplemented with IPTG 200 µM for functional complementation.
[b] cryptic growth (*i.e.* non exponential growth and low biomass final titer, see **Figure 1A**).
