# Supplementary figures and images for: Molecular and Physiological Logics of the Pyruvate-Induced Response of a Novel Transporter in Bacillus subtilis
Source: mBio. 2017 Oct 3;8(5):e00976-17. doi: 10.1128/mBio.00976-17 (PMC5626966; doi:10.1128/mBio.00976-17)

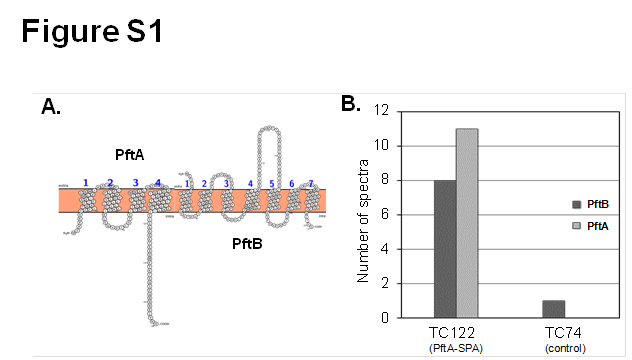

Supplement: FIG S1 [file mbo005173508sf1.tif]

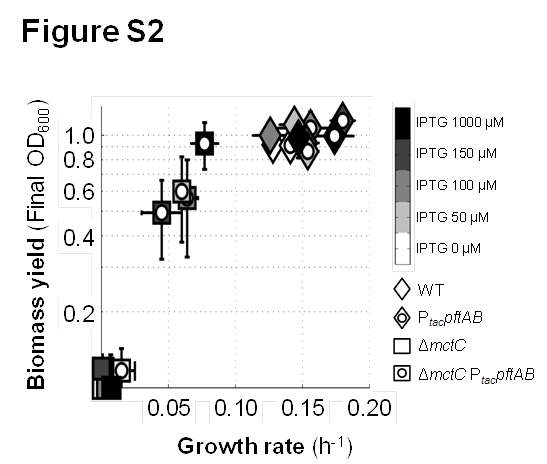

Supplement: FIG S2 [file mbo005173508sf2.tif]

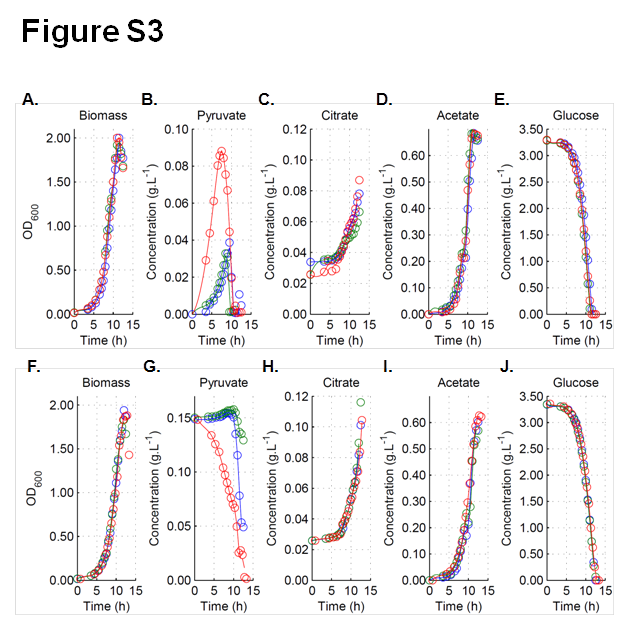

Supplement: FIG S3 [file mbo005173508sf3.tif]

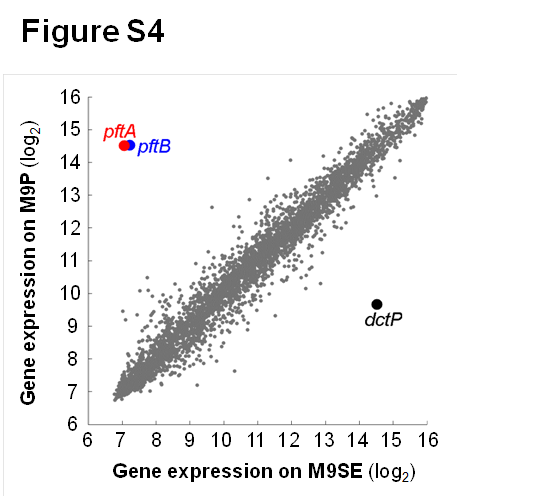

Supplement: FIG S4 [file mbo005173508sf4.tif]

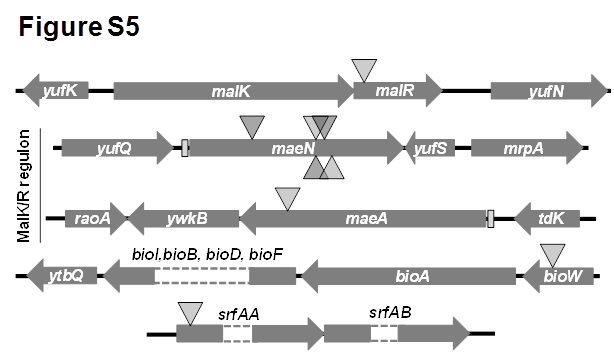

Supplement: FIG S5 [file mbo005173508sf5.tif]

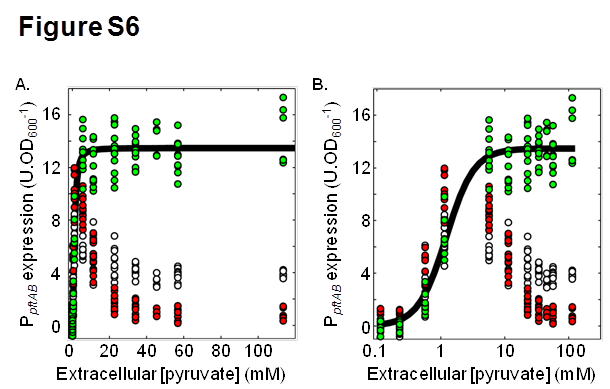

Supplement: FIG S6 [file mbo005173508sf6.tif]
