# Supplementary material for: Molecular and Physiological Logics of the Pyruvate-Induced Response of a Novel Transporter in Bacillus subtilis
Source: mBio. 2017 Oct 3;8(5):e00976-17. doi: 10.1128/mBio.00976-17 (PMC5626966; doi:10.1128/mBio.00976-17)
Supplement: TEXT S1 [file mbo005173508s1.docx]

**Supplemental Text S1**

**for**

**Molecular and physiological logics of the intra- and extracellular pyruvate-induced response of a novel transporter in *Bacillus subtilis***

Teddy Charbonnier^1^, Dominique Le Coq^1,2^, Stephen McGovern^1^, Magali Calabre^1^, Olivier Delumeau^1^, Stéphane Aymerich^1^ and Matthieu Jules^‡1^

*^1^ Micalis Institute, INRA, AgroParisTech, Université Paris-Saclay, 78350 Jouy-en-Josas, France; ^2^ Micalis Institute, INRA, AgroParisTech, CNRS, Université Paris-Saclay, 78350 Jouy-en-Josas, France.*

^‡^ corresponding author

**Running title:** Pyruvate transport and regulation in *B. subtilis*

**Table of content**

[1 Supplemental Materials and Methods 3](#_Toc484600209)

[a. Tandem affinity purification 3](#_Toc484600210)

[b. In-gel digestion and nanoLC-MS/MS analysis 3](#_Toc484600211)

[c. Proteomic data processing and bioinformatics 4](#_Toc484600212)

[d. His_6_-CcpA, His_6_-LytT and His_6_-LytS production and purification for EMSA 5](#_Toc484600213)

[2 Supplemental Results 6](#_Toc484600214)

[a. Kinetic properties of PftAB 6](#_Toc484600215)

[b. Modeling the induction of *pftAB* expression by LytST 8](#_Toc484600216)

[c. Modeling the putative intracellular pyruvate-mediated repression via LytST 9](#_Toc484600217)

[3 Supplemental References 11](#_Toc484600218)

# Supplemental Materials and Methods

## Tandem affinity purification

As the tandem affinity purification (TAP-tag) technique has been developed to study protein-protein interactions, we used the PftA-SPA and PftB-SPA expressing strains to perform similar experiment. In the chromosome of *B. subtilis* *pft*A and *pft*B are adjacent, and the translational fusion of *pft*A and the SPA sequence was obtained by the integration of the whole plasmid (pMUTIN-SPALIC derivatives) by single cross-over. In this construct, an IPTG-inducible promoter (strain TC122, **Table 1**) was inserted right upstream of *pftB* to ensure that the gene is expressed. This prompted the question whether PftB would be expressed at a high level and become a contaminant after PftA-SPA purification by tandem affinity. As a control, we used a strain that expressed *pftA* and *pftB* under the control of the IPTG inducible P*_hs_* promoter (strain TC74, **Table 1**) and the minimal IPTG concentration for inducing PftA and PftB at a level able to sustain growth on pyruvate was determined. This was obtained with 50 µM IPTG. Therefore strains TC74 and TC122 were grown in M9P + 50 µM IPTG. The procedure for tandem affinity purification was conducted in parallel for the two strains, proteins were separated on gel and the purified proteins were identified by mass spectrometry (see below).

## In-gel digestion and nanoLC-MS/MS analysis

Each lane of the gel was cut and washed for 15 min with an acetonitrile / 100 mM ammonium bicarbonate mixture (1:1). Digestion was performed in 50 mM ammonium bicarbonate pH 8.0 and the quantity of modified trypsin (Promega, sequencing grade) was 0.1 μg per sample. Digestion was achieved for 6 h at 37°C. A second digestion with chymotrypsin (Roche, sequencing grade) was achieved: 0.1 μg per sample. The supernatant was conserved. Peptides were extracted by 5% formic acid in water/acetonitrile (v/v). Supernatant and extract tryptic peptides were dried and resuspended in 20 μl of 0.1% (v/v) trifluoroacetic acid.

HPLC was performed on an Dionex RSLCnano (Thermo Fisher Scientific). A 4 µL sample was loaded at 20 µL/min-1 on a precolumn cartridge (stationary phase: C18 PepMap 100, 5 µm; column: 300 µm i.d., 5 mm; Dionex) and desalted with 0.08% TFA and 2% acetonitrile. After 4 min, the precolumn cartridge was connected to the separating PepMap C18 column (stationary phase: C18 PepMap 100, 3 µm; column: 75 µm i.d., 150 mm; Dionex). Buffers were 0.1% formic acid, 2% acetonitrile (A) and 0.1% formic acid and 80% acetonitrile (B). The peptide separation was achieved with a linear gradient from 0 to 36% B for 30 min at 300 nL/min-1. Including the regeneration step at 100% B and the equilibration step at 100% A, one run took 42 min. Eluted peptides were analysed on-line with a LTQ-Orbitrap mass spectrometer (Thermo Electron) using a nanoelectrospray interface. Ionization (1.3 kV ionization potential) was performed with liquid junction and a capillary probe (10 µm i.d.; New Objective). Peptide ions were analysed using Xcalibur 2.07 with the following data-dependent acquisition steps (1): full MS scan in orbitrap (mass-to-charge ratio (m/z) 300 to 1600, profil mode) and (2) MS/MS in linear trap (qz = 0.25, activation time = 30 ms, and collision energy = 45%; centroid mode). Step 2 was repeated for the eight major ions detected in step 1. Dynamic exclusion time was set to 30 s.

## Proteomic data processing and bioinformatics

The *Bacillus subtilis* 168 database was downloaded from UniprotKB Database site (March 2015, 4242 protein entries). In addition, the sequence of PftA-SPA was incorporated in this database and in conjunction with reverse and contaminant databases, were searched by X!Tandem (version 2013.09.01.1, <http://www.thegpm.org/tandem/>) using X!Tandempipeline (version 3.3.4) developed by PAPPSO (<http://pappso.inra.fr/bioinfo/>). Enzymatic cleavage was declared as a trypsin and a chymotrypsin digestion with one possible misscleavage. Cys carboxyamidomethylation and Met oxidation were set to static and possible modifications, respectively. Precursor mass was 10 ppm and fragment mass tolerance was 0.02 Da. Only peptides with a E value smaller than 0.1 were reported. TandemPipeline (<http://pappso.inra.fr/bioinfo/xtandempipeline/>) according to: (1) A minimum of two different peptides was required with an E value smaller than 0.05, (2) a protein log (E value) (calculated as the product of unique peptide E values) smaller than 2.10^-3^. These criteria led to a False Discovery Rate (FDR) of 0.05 % for peptide and protein identification. To take redundancy into account, proteins with at least one peptide in common were grouped. This allowed to group proteins of similar function. Within each group, proteins with at least one specific peptide relatively to other members of the group were reported as sub-groups. Although the analysis by mass spectrometry revealed various contaminants we focused our analysis on the presence of PftB (**Figure S1B**).

## His_6_-CcpA, His_6_-LytT and His_6_-LytS production and purification for EMSA

Cells were grown at 30°C in 1 L LB medium containing 30 µg.mL^-1^ kanamycin (expression of His_6_-LytT and His_6_-LytS) or 100 µg.mL^-1^ ampicillin (expression of His_6_-CcpA). Expression was induced for 3 hours by the addition of 500 µM IPTG when biomass reached an OD_600_ of 0.7. Cells were harvested by centrifugation, and the pellet was resuspended in 40 mL 50 mM Tris-HCl pH 8.0, 1 M NaCl and stored at -20°C. Cells were lysed by sonication and centrifuged at 100000 g for 1 hour at 4°C. Supernatant containing the His-tagged proteins were loaded onto a Ni^2+^ affinity column (Ni-NTA agarose, Qiagen) pre-equilibrated in the same buffer. The Ni-NTA columns were washed with 100 mL 50 mM Tris-HCl pH 8.0, 1 M NaCl and then with 50 mL 50 mM Tris-HCl pH 8.0, 1 M NaCl, 5 mM imidazole. After washing again with 10 mL of buffer 50 mM Tris-HCl pH 8.0, 0.4M NaCl, 20 mM Imidazole, proteins were eluted with the same buffer supplemented with 250 mM imidazole and dialyzed against 50 mM Tris-HCl pH 8.0, 0.4 M NaCl, 50 % glycerol, 1 mM DTT, prior to storage at -20°C. An additional step was added in order to separate His_6_-LytT from remaining contaminating proteins. The dialyzed fraction obtained after elution from Ni-resin (1 ml) was diluted in 9 mL 50 mM Tris-Hcl pH 8.0, and loaded onto a 1 mL Hitrap Heparin column (GE) equilibrated in 40 mM NaCl, 50 mM Tris-HCl pH 8.0. The contaminants were found in the flow-through, while the His_6_-LytT protein was eluted by applying a gradient in 15 mL 50 mM Tris-HCl pH 8.0 from 40 mM NaCl to 1 M NaCl. The His_6_-LytT protein containing fraction was again dialyzed against 50 mM Tris-HCl pH 8.0, 0.4 M NaCl, 50 % glycerol, 1 mM DTT, prior to storage at -20°C.

# Supplemental Results

## Kinetic properties of PftAB

*General mathematical framework for a simple pore (Facilitated transport)*

For a simple pore (**Figure below** from Pradhan *et al.* (51)), the set of equations describing the rates of change of bound and unbound states of the protein are:

$\frac{\boldsymbol{d}\mathbf{[}\boldsymbol{C}\boldsymbol{S}_{\mathbf{1}}\mathbf{]}}{\boldsymbol{dt}}\mathbf{=}\boldsymbol{k}_{\mathbf{1}}\left[ \boldsymbol{C} \right]\left[ \boldsymbol{S}_{\mathbf{1}} \right]\mathbf{-}\boldsymbol{k}_{\mathbf{-1}}\left[ \boldsymbol{C}\boldsymbol{S}_{\mathbf{1}} \right]{\mathbf{-}\boldsymbol{k}}_{\mathbf{2}}\left[ \boldsymbol{C}\boldsymbol{S}_{\mathbf{1}} \right]\mathbf{+}\boldsymbol{k}_{\mathbf{-2}}\mathbf{[}\boldsymbol{C}\boldsymbol{S}_{\mathbf{2}}\mathbf{]}$ {a.1}
$\frac{\boldsymbol{d}\mathbf{[}\boldsymbol{C}\boldsymbol{S}_{\mathbf{2}}\mathbf{]}}{\boldsymbol{dt}}\mathbf{=}\boldsymbol{k}_{\mathbf{2}}\left[ \boldsymbol{C}\boldsymbol{S}_{\mathbf{1}} \right]\mathbf{-}\boldsymbol{k}_{\mathbf{-2}}\left[ \boldsymbol{C}\boldsymbol{S}_{\mathbf{2}} \right]{\mathbf{-}\boldsymbol{k}_{\mathbf{3}}\mathbf{[}\boldsymbol{C}\boldsymbol{S}_{\mathbf{2}}\mathbf{]+}\boldsymbol{k}}_{\mathbf{-3}}\left[ \boldsymbol{C} \right]\left[ \boldsymbol{S}_{\mathbf{2}} \right]$ {a.2}
$\left[ \boldsymbol{C} \right]\mathbf{=}\left[ \boldsymbol{C}_{\boldsymbol{tot}} \right]\mathbf{-}\left[ \boldsymbol{C}\boldsymbol{S}_{\mathbf{1}} \right]\mathbf{-}\left[ \boldsymbol{C}\boldsymbol{S}_{\mathbf{2}} \right]$ {a.3}

where the solute S in aqueous phase 1 (S1; *eg* extracellular) is translocated to aqueous phase 2 (S2; *eg* intracellular) by first associating with the free carrier (C) forming a solute carrier complex CS1. CS1 undergoes a conformational change to CS2, which dissociates and releases solute S to aqueous phase 2 (S2). The rate constants for each elementary step *i* are denoted by *k*_i_ for the forward direction and *k*_-i_ for the reverse direction.

Under steady-state operation of the catalytic cycle described above and in equations {a.1-3}, it is straightforward to show that the net solute flux through the pore is given by:

$J_{net}=k_{2}\left[ CS_{1} \right]-k_{-2}\left[ CS_{2} \right]=\left[ C_{tot} \right]\frac{\left( k_{1}k_{2}k_{3}\left[ S_{1} \right]-k_{-1}k_{-2}k_{-3}\left[ S_{2} \right] \right)}{D}$ {a.4}
with the denominator,

$$D=k_{-1}\left( k_{-2}+k_{3} \right)+k_{2}k_{3}+k_{1}\left( k_{-2}+k_{2}+k_{3} \right)\left[ S_{1} \right]+k_{-3}\left( k_{-2}+k_{-1}+k_{2} \right)\left[ S_{2} \right]$$

At equilibrium, the net solute flux through the carrier is zero, which results in the following thermodynamic constraint for the rate constants governing the simple pore model:

$\frac{\left[ S_{1} \right]^{eq}}{\left[ S_{2} \right]^{eq}}=\frac{k_{1}k_{2}k_{3}}{k_{-1}k_{-2}k_{-3}}$ {a.5}
 Under rapid equilibrium assumption, it is straightforward to show that the net solute flux through the pore is given by:

$J_{net}=k_{2}\left[ CS_{1} \right]-k_{-2}\left[ CS_{2} \right]=\left[ C_{tot} \right]\frac{\left( k_{2}\left[ S_{1} \right]-k_{-2}\left[ S_{2} \right] \right)}{D}$ {a.6}
with the denominator, $D=K_{s}+\left[ S_{1} \right]+\left[ S_{2} \right]$, $K_{s}$ being the dissociation equilibrium constants. The equilibrium relationship and the thermodynamic constraint for the rate constants governing the simple pore model (equation {a.6}) is then reduced to:

$\frac{\left[ S_{1} \right]^{eq}}{\left[ S_{2} \right]^{eq}}=\frac{k_{2}}{k_{-2}}$ {a.7}

*Estimation of the V_M_ (J_max_) and K_M_ of PftAB*

For *B. subtilis* grown on M9G (growth condition **(4)** from **Table S2**), $\frac{k_{2}}{k_{-2}}\sim1$ (${[Pyr]}_{in}^{eq}$ is of about 1 mmol.L^-1^ on M9G (26) when ${[Pyr]}_{ex}^{eq}$ reached a maximum at about 1 mmol.L^-1^, **Figure 2** and **Figure S3**). Under the assumption that $\frac{{[Pyr]}_{in}^{eq}}{{[Pyr]}_{ex}^{eq}}=\frac{k_{2}}{k_{-2}}\sim1$, it is straightforward to show that the net pyruvate flux in a given growth condition ($J_{net}^{(i)})$ is:

$J_{net}^{(i)}=\frac{J_{max}({[Pyr]}_{ex}^{(i)}-{[Pyr]}_{in}^{(i)})}{K_{M}+{[Pyr]}_{ex}^{(i)}+{[Pyr]}_{in}^{(i)}}$ {a.8}
where ${[Pyr]}_{ex}^{(i)}$ and ${[Pyr]}_{in}^{(i)}$ are the extracellular and intracellular concentrations of pyruvate in the growth condition (*i*); $J_{max}=k_{2}\left[ YsbAB \right]_{\mathrm{Tot}}$ is the maximal transport rate; and $K_{M}$ is the Michaelis–Menten constant for transport. We can rewrite equation {a.8} as follows:

$K_{M}=\frac{J_{max}({[Pyr]}_{ex}^{(i)}-{[Pyr]}_{in}^{(i)})-J_{net}^{(i)}({[Pyr]}_{ex}^{(i)}+{[Pyr]}_{in}^{(i)})}{J_{net}^{(i)}}$ {a.9}
$J_{max}$ depending on the level of PftAB, we have chosen to normalize the expression of *pftAB* in the different growth conditions to the growth condition M9P (growth condition **(2)** from **Table S2**) using the *pftAB* expression levels. Hence, $J_{max}$ corresponds from now on to the maximal transport rate of cells grown in presence of pyruvate as sole carbon source (*i.e.* for a level of expression of PftAB of about 4.5 U.OD_600_^-1^).

Making use of the physiological parameters and of the levels of expression of PftAB in the different growth conditions (**Table S2** and **Figure next page**), we next solved equation {a.9} and inferred the $J_{max}$ and $K_{M}$ using the following constraints:

- -$J_{max}>J_{net}^{(2)}>0$
  - ${[Pyr]}_{in}^{(2)}<{[Pyr]}_{out}^{(2)}$
  - ${{[Pyr]}_{in}^{(5)}\sim{[Pyr]}_{in}^{(6)}>[Pyr]}_{in}^{(2)}>{[Pyr]}_{in}^{(4)}\sim{[Pyr]}_{in}^{(1)}>0$

**The resolution of the abovementioned constrained equations led to** $\boldsymbol{J}_{\boldsymbol{max}}\boldsymbol{=10.0}\boldsymbol{\pm}\boldsymbol{1.0}$ **mmol.h^-1^.gCDW^-1^ (relative to the level of PftAB in M9P) and** $\boldsymbol{K}_{\boldsymbol{M}}\boldsymbol{=1.0}\boldsymbol{\pm}\boldsymbol{0.1}$ **mmol.L^-1^.**

**Expression of P*_hs_gfp* and P*_pftAB_gfp*** in M9P, M9SE and M9G were used to infer the kinetic properties of PftAB in relation with the measured level of expression of *pftAB* (**Table S1**). Mean values of at least six experiments with standard deviations are presented.

## Modeling the induction of *pftAB* expression by LytST

As recently shown by Wei *et al* (2014), the active response regulator concentration ([RP]) of a classical TCS increases linearly in proportion to the signal strength (*i.e.* the amount of inducer/ligand, [L]) (38), so that we can write:

$[RP]=\alpha\left[ L \right]$ {b.1}
Note that this relationship remains true only until the response regulator is fully activated. Afterwards $\left[ \mathrm{RP} \right]$ remains constant (*i.e.* 100% of total response regulator).

In the Δ*pftAB* strain, we can reasonably assume that the uptake of pyruvate by cells grown in M9SE+P is close to zero and therefore that the intracellular pyruvate concentration is comparable to that in M9SE. Hence, the LytST TCS-mediated induction of P*_pftAB_* must not be affected by the level of intracellular pyruvate and behave as for the classical TCS so that: $\left[ \mathrm{LytT}_{\mathrm{act}} \right]=\alpha\left[ L \right]$ with $\mathrm{LytT}_{\mathrm{act}}$ and L corresponding to the active LytT and extracellular pyruvate, respectively.

Wei *et al* (24) also developed a deterministic model of TCS induction in which the proportion of time for which DNA is activated is represented by the Hill function (38):

$P^{\left( \mathrm{active} \right)}=\frac{{[RP]}^{n}}{{\mathrm{Kd}^{n}+[RP]}^{n}}$ {b.2}
 where Kd is the microscopic dissociation constant of RP to its regulatory site, n is the multimerisation of RP required to bind to DNA (most generally n=2).

Considering for LytT a simple model of TCS-mediated promoter induction by a dimeric response regulator (equation {b.1} into {b.2} with n=2) leads to:

$P_{ysbAB}^{\left( \mathrm{active} \right)}=\frac{{[\mathrm{LytT}_{\mathrm{act}}]}^{2}}{\mathrm{Kd}^{2}+{[\mathrm{LytT}_{\mathrm{act}}]}^{2}}=\frac{{(\alpha[L])}^{2}}{\mathrm{Kd}^{2}+{(\alpha[L])}^{2}}$ {b.3}
In steady-state growth, we can derive the level of GFP abundance:

$\frac{\partial GFP}{\partial t}=k_{0}+{k_{1} P}_{ysbAB}^{\left( \mathrm{active} \right)}-\mu\left[ \mathrm{GFP} \right]-k_{deg}\left[ \mathrm{GFP} \right]=0$ {b.4}
 with $k_{0}$ and $k_{1}$ the basal production rates, *µ* the growth rate and $k_{deg}$ the GFP degradation rate. Note that $k_{0}=0$ since the promoter of *pftAB* is off in the absence of induction and $k_{deg}$ is negligible (for the very stable GFPmut3 variant used in this work, (43)) as compared to $\mu$. Equation {b.3} into {b.4} therefore simplifies into:

$\left[ \mathrm{GFP} \right]=\frac{k_{1}}{\mu}P_{ysbAB}^{\left( \mathrm{active} \right)}=\frac{k_{1}}{\mu}\left( \frac{{[\mathrm{LytT}_{\mathrm{act}}]}^{2}}{\mathrm{Kd}^{2}+{[\mathrm{LytT}_{\mathrm{act}}]}^{2}} \right)=\frac{k_{1}}{\mu}\left( \frac{{(\alpha[L])}^{2}}{\mathrm{Kd}^{2}+{(\alpha[L])}^{2}} \right)$ {b.5}
which can be further written as:

$\left[ \mathrm{GFP} \right]=\frac{k_{s}}{\mu}\left( \frac{{[L]}^{2}}{\mathrm{Kd}_{a}+{[L]}^{2}} \right)$ {b.6}
 where $\mathrm{Kd}_{a}=\left( \frac{\mathrm{Kd}^{2}}{\alpha^{2}} \right)$ and $k_{s}=k_{1}$

Plotting the data of the Δ*pftAB* strain according to the Hill equation with a coefficient of 2 revealed a maximal activity ($\frac{k_{s}}{\mu})$ of ~7000 s^-1^ and an apparent dissociation constant ($\mathrm{Kd}_{a}$) of ~1.5 (**Figure S6**). However, note that by construction the above-mentioned Hill equation reaches a plateau when $[L]$ is saturating and cannot therefore fit the data for the wild-type and P*_hs_pftAB* strains from **Figure S6**.

## Modeling the putative intracellular pyruvate-mediated repression via LytST

In order to get insight into the mechanism of intracellular pyruvate-mediated "repression" via LytST, we modeled three types of putative inhibitions of LytT activation (*i.e.* phosphorylation) by intracellular pyruvate (I): competitive inhibition, uncompetitive inhibition and non competitive inhibition. We solved the related differential equations at steady state and obtained for:

- competitive inhibition: $\left[ \mathrm{LytT}_{\mathrm{act}} \right]=\frac{\mathrm{LytT}_{\mathrm{total}} [L]}{K_{M} \left( 1+\frac{[I]}{Ki} \right)+[L]}$ {c.1}
- uncompetitive inhibition: $[\mathrm{LytT}_{\mathrm{act}}] =\frac{\mathrm{LytT}_{\mathrm{total}} [L]}{K_{M}+\left( 1+\frac{[I]}{Ki} \right) [L]}$ {c.2}
- non competitive inhibition: $\left[ \mathrm{LytT}_{\mathrm{act}} \right]= \frac{\mathrm{LytT}_{\mathrm{total}} [L]}{\left( 1+\frac{\left[ I \right]}{Ki} \right) \left( K_{M}+[L] \right)}$ {c.3}

$\mathrm{LytT}_{\mathrm{total}}$ corresponds to the total amount of LytT (active+inactive); L and I correspond to extracellular and intracellular pyruvate, respectively; $K_{M}$ corresponds somehow to the affinity constant for LytS (under the assumption that $\left[ \mathrm{LytS}_{\mathrm{act}} \right]$ is linearly correlated with [L]); and $Ki$ corresponds to the inhibition constant.

We were unable to identify relevant parameters that can fit the data from **Figure S6** for the wild-type and P*_hs_pftAB* strains using these three equations. Indeed, introducing equations {c.1} to {c.3} into equation {b.2} (with n=2) directly reveals that in order to observe a repression (*i.e.* reduction of GFP abundance when the level of extracellular pyruvate increases) the equations {c.1} to {c.3} must be minimized. By construction minimizing equation {c.1} implies that [I] decreases when [L] increases. The conclusion is similar for equations {c.2} and {c.3}. These conclusions are not consistent with the assumption that intracellular pyruvate hinders LytST induction (since we expect that an increase of extracellular pyruvate will lead to an increase of intracellular pyruvate). We therefore concluded that none of the modeled inhibitions of LytT activation can explain the reduction of the LytST-mediated induction of *pftAB*.

Different other molecular mechanisms may however explain the feed-back regulation of LytST by intracellular pyruvate (and/or an intermediate of overflow metabolism), such as an inhibition of LytS autophosphorylation or a hindered recruitment of the RNA polymerase.

# Supplemental References

49. van Dijl JM, Dreisbach A, Skwark MJ, Sibbald MJJB, Tjalsma H, Zweers JC, Buist G. 2012. Ins and Outs of the *Bacillus subtilis* Membrane Proteome, p 253-283. *In* Graumann P (ed), *Bacillus* : Cellular and Molecular Biology. Caister Academic Press, Wymondham.

50. Omasits U, Ahrens CH, Muller S, Wollscheid B. 2014. Protter: interactive protein feature visualization and integration with experimental proteomic data. Bioinformatics 30:884-6.

51. Pradhan RK, Vinnakota KC, Beard DA, Dash RK. 2013. Chapter 5 - Carrier-Mediated Transport Through Biomembranes, p 181-212. *In* Becker SM, Kuznetsov AV (ed), Transport in Biological Media. Elsevier, Boston.
