# Supplementary material for: Molecular and Physiological Logics of the Pyruvate-Induced Response of a Novel Transporter in Bacillus subtilis
Source: mBio. 2017 Oct 3;8(5):e00976-17. doi: 10.1128/mBio.00976-17 (PMC5626966; doi:10.1128/mBio.00976-17)
Supplement: TABLE S2 [file mbo005173508st2.pdf]

Table S2. Pyruvate concentrations, specific pyruvate uptake rate and PftAB expression level in various growth conditions

Growth conditions "(*i*)"	**(1)**	**(2)**	**(3)**	**(4)**	**(5)**	**(6)**
Medium	MM+G	M9P	M9G+P	M9G	M9PM	M9M
$J_{net}^{(i)} = q_{pyr}$ (mmol.h^{-1}.gCDW^{-1})	-	8.26 ± 0.23 [f]	1.90 ± 0.06 [c]	-5.00 ± 0.15 [c]	0.90 ± 0.10 [g]	-
$[Pyr]_{ex}^{(i)}$ (mmol.L^{-1})	-	68.14 ± 0.07 [f]	1.70 ± 0.01 [c]	0 [a]	45.42 ± 0.07 [g]	-
$[Pyr]_{in}^{(i)}$ (mmol.L^{-1})	1.00 ± 0.20 [e]	*n.d.* [b]	*n.d.* [b]	*n.d.* [b]	*n.d.* [b]	10.00 ± 2.00 [h]
pftAB under the control of	P_{pftAB}	P_{pftAB}	P_{hs}	P_{hs}	P_{pftAB}	P_{pftAB}
PftAB expression level (U.OD^{-1})	0.00 ± 0.10 [d]	4.50 ± 0.45 [d]	4.50 ± 0.50 [d]	4.50 ± 0.50 [d]	0.50 ± 0.10 [d]	0.70 ± 0.15 [d]

[a] At the beginning of the growth (initial condition)
[b] *n.d.* not determined
[c] this work, inferred from the data plotted on **Figure S3**
[d] this work (**Table 3** and in **Supplemental Results**)
[e] from reference (26)
[f] from reference (7)
[g] from reference (12)
[h] from reference (22)
