# Supplementary material for: Molecular and Physiological Logics of the Pyruvate-Induced Response of a Novel Transporter in Bacillus subtilis
Source: mBio. 2017 Oct 3;8(5):e00976-17. doi: 10.1128/mBio.00976-17 (PMC5626966; doi:10.1128/mBio.00976-17)
Supplement: TABLE S3 [file mbo005173508st3.pdf]

Table S3. Relationships between *B. subtilis* and *C. glutamicum* strains, plasmids and primers used in this work

Strain	Plasmid	Primer	Sequence (5'→3') [a]	Template
TC35	pBSB_P$_{pftAB}$	P$_{pftAB}$_FW	CCGCGGGCTTTCCCAGCCTCTTGATTATTTAACCAA	BSB168
		P$_{pftAB}$_RV	GTTCCTCCTTCCCACCCCTCATTATAAATAAGAA	BSB168
TC58	pGEMT_*lytST*	*LytT*_FW	GATAAAGGAAGCGCAATTGC	BSB168
		*LytT*_RV	CAATGGAACAGGCAGTTTTA	BSB168
	pGEMT_*lytST::cm*	*Cm_lytT*_FW	TCCCCCGGGGGATTCACCGACAAACAACAGATAAAA	pDG1661
		*Cm_lytT*_RV	CCATCGATGGTATAAAAACCTTCTTCAACTAACGGG	pDG1661
TC63	pDG1661_P$_{pftAB}$	P$_{pftB}$_FW	CGGGATCCCGTTTGGTACCCTCATTATAAATAAGAA	BSB168
		P$_{pftB}$_RV	CCCAAGCTTGGGGCTTTGTCGTGAATACTGAATATA	BSB168
TC60, TC61 & TC62	pGEMT_*pftAB*	insert_*pftAB*_FW	CCCCACCCAGAATACAAAGA	BSB168
		insert_*pftAB*_RV	GGATGAACTGGCTTACTTGC	BSB168
	pGEMT_*pftA*	pGEMT_*pftA*_FW	TCCCCCGGGGGAAATCATATTTGAAACCAGCATAAT	pGEMT_*pftAB*
		pGEMT_*pftA*_RV	GCTCTAGAGCACAAAAACAGTGCAAAGCCCGCAGAA	pGEMT_*pftAB*
	pGEMT_*pftB*	pGEMT_*pftB*_FW	TCCCCCGGGGGAACCATGGCAACAAACAAAGGAGTGA	pGEMT_*pftAB*
		pGEMT_*pftB*_RV	GCTCTAGAGCTAAAGGATTGGCTCTTGGAACATCCG	pGEMT_*pftAB*
	pGEMT_*pftAB::cm*	*cm*_FW	TCCCCCGGGGGATCTTTATTCTTCAACTAAAGCACCCATTAG	pDG1661
	pGEMT_*pftB::cm*	*cm*_RV	GCTCTAGAGCGTAGAGGATCTGGAGCTGTA	pDG1661
	pGEMT_*pftA::cm*_P$_{pftAB}$	*cm*_P$_{pftAB}$_FW	GCTCTAGAGCGGATCCCGTTTGGTACCCTCATTATA	pDG1661_P$_{pftA}$
		*cm*_P$_{pftAB}$_RV	TCCCCCGGGGGAATAAAAACCTTCTTCAACTAACGG	pDG1661_P$_{pftA}$
TC73	pMUTIN4_*lytST*	*lytST*_FW	CGGGATCCATATTTTTTCTCCGCCTGTAAGCAAGTT	BSB168
		*lytST*_RV	CGAGCTCGTTATAAAGCGCTTACAAGATAACATGCC	BSB168

TC74, TC75 & TC76	pDR111_*pftAB*	*pftAB*_FW	TCACGCGTCGACAGGAGGACTACTATGAGTGCTAAAAAAGTGTAC	BSB168
		*pftB*_RV	TCCTAGCTAGCTTATCCTCCGATCAGCTGCACGAACAC	BSB168
TC153, TC154	pDR111_*pftAB*	618 FW	AAGGAAAAAAGCGGCCGCCAAACTTAAGAGTGTGTT	pic618
		618 RV	CTGGTGAAAAGAAAAACCACCCTGGCGCCCAATACG	pic618
	pDG1664_P_{hs}*pftAB*	P_{hs}*pftAB* FW	AAATACGGGCAGACATGGCCTGCCCGGTTATTATTA	pDR111_*pftAB*
		P_{hs}*pftAB* RV	AAGGAAAAAAGCGGCCGCGATTAATTAATTCAGAACGCTCGGTTG	pDR111_*pftAB*
TC125, TC126 & TC149	pBSB_$P_{pftAB\text{-}lytT1}$,	*lytT*1_FW	CGGGATCCATTCTGCAACTCAGACCCGCTATACG	pBSB_P_{pftAB}
	pBSB_$P_{pftAB\text{-}lytT2}$	*lytT*1,2_FW	CGGGATCCAGACCCGCTATACGGCATGTTATCT	pBSB_P_{pftAB}
	& pBSB_$P_{pftAB\text{-}lytT1.2}$	*lytT*2_RV	GCGGATCCCGCAAATGGCTGAAATGCAA	pBSB_P_{pftAB}
		*lytT*1,2_RV	GCGGATCCCGAAAAAGCCGCCCATCAAATATGG	pBSB_P_{pftAB}
TC122, TC124	pPftA_SPA	*pftA*-LIC1	GGGTTCCTGGCGCGAGCCGGGTTTTTAACACAAGC	BSB168
		pftA-LIC2	TTGGGCTGGCGCGAGCATGGTGAACGAGTTCGTTATTG	BSB168
	pPftB_SPA	*pftB*-LIC1	GGGTTCCTGGCGCGAGCGGCTCTATCTGCTCTGTCACG	BSB168
		pftB-LIC2	TTGGGCTGGCGCGAGCTCCTCCGATCAGCTGCACG	BSB168
TC199	pSG-SPA-NTER	OOD141	GGGCTCGAGATGAGTGCTAAAAAAGTGTACGGG	BSB168
		OOD142	GGGGCGGCCGCTTAATGGTGATGGTGATGGTGTCCTCCGATCAGCTGCACG	BSB168
TC202, TC203	pXMJ_*pftAB*	*pftAB_Cg_fw*	TCACGCGTCGACAGGAGGACTACTATGAGTGCTAAAAAAGTGTAC	BSB168
		pftAB_Cg_rev	TCCCCCCGGGGTTATCCTCCGATCAGCTGCACGAACAC	BSB168

[a] Restriction sites and additional LIC and His_6 sequences are underlined
